# Chd8 regulates X chromosome inactivation in mouse through fine-tuning control of *Xist* expression

**DOI:** 10.1038/s42003-021-01945-1

**Published:** 2021-04-15

**Authors:** Andrea Cerase, Alexander N. Young, Nerea Blanes Ruiz, Andreas Buness, Gabrielle M. Sant, Mirjam Arnold, Monica Di Giacomo, Michela Ascolani, Manish Kumar, Andreas Hierholzer, Giuseppe Trigiante, Sarah J. Marzi, Philip Avner

**Affiliations:** 1 EMBL-Rome, Epigenetics and Neurobiology Unit, Monterotondo, Italy; 2 Blizard Institute, Barts and The London School of Medicine and Dentistry, Queen Mary University of London, London, UK; 3 Core Unit for Bioinformatics Data Analysis Universitätsklinikum Bonn, Bonn, Germany; 4 Institute of Molecular Biology gGmbH (IMB), Mainz, Germany; 5 Max Planck Institute for Molecular Genetics, Otto Warburg Laboratory, Berlin, Germany; 6 Department of Allied Health Science, Shri B. M. Patil Medical College, Hospital and Research Centre, BLDE, Vijaypura, Karnataka India; 7 UK Dementia Research Institute, Imperial College London, London, UK; 8 Present Address: Department of Biosystems Science and Engineering, ETH Zürich, Basel, Switzerland

**Keywords:** Stem cells, Chromatin, Epigenetics, Long non-coding RNAs

## Abstract

Female mammals achieve dosage compensation by inactivating one of their two X chromosomes during development, a process entirely dependent on *Xist*, an X-linked long non-coding RNA (lncRNA). At the onset of X chromosome inactivation (XCI), *Xist* is up-regulated and spreads along the future inactive X chromosome. Contextually, it recruits repressive histone and DNA modifiers that transcriptionally silence the X chromosome. *Xist* regulation is tightly coupled to differentiation and its expression is under the control of both pluripotency and epigenetic factors. Recent evidence has suggested that chromatin remodelers accumulate at the X Inactivation Center (*XIC*) and here we demonstrate a new role for Chd8 in *Xist* regulation in differentiating ES cells, linked to its control and prevention of spurious transcription factor interactions occurring within *Xist* regulatory regions. Our findings have a broader relevance, in the context of complex, developmentally-regulated gene expression.

## Abstract (web summary)

Andrea Cerase et al. report that the chromatin remodeler Chd8 is a key regulator of mammalian *Xist* expression and therefore X chromosome inactivation. They find that Chd8 activates *Xist* expression in embryonic stem cells, while in differentiating cells it acts to prevent spurious *Xist* expression through blocking transcription factor binding to the *Xist* promoter.

## Introduction

In mammals, sex is determined by the presence, number and combination of X and Y chromosomes^1–4^. Female mammals silence one of their two X chromosomes early in development in order to ensure X-linked gene dosage between females and males^1,2^. In mouse, an imprinted form of this process, where it is always the paternal X that is inactivated, is initiated in early development at the 2–4-cell stage (imprinted XCI). This form of XCI is then reversed during embryonic development at the blastocyst stage in the cells of the inner cell mass (ICM), where both X-chromosomes become active and subsequently have equal chances to be inactivated during the onset of gastrulation^5^. *Xist* lncRNA is the master regulator of XCI^6^. Its mono-allelic upregulation and *in cis-*spreading of *Xist* RNA triggers the initiation of the inactivation process by recruitment, directly or indirectly, of repressive chromatin and of DNA modifiers to silence gene-transcription^6–13^.

Differentiating female ES cells, carrying two X chromosomes, are a widely used model for the study of X chromosome inactivation as they closely recapitulate the early phases of random XCI^4,14–16^ in the embryo, and have been instrumental in allowing the dissection of the molecular pathways involved in *Xist* regulation. Previous work from the Avner laboratory has demonstrated that transcription of *Xist* and the *Tsix* lncRNA, its major antagonist, is precisely controlled by pluripotency factors^17–19^. Other laboratories have investigated the role of nearby genes encoding lncRNAs such as *Jpx* and *Ftx* and protein factors such as *Rnf12* (also known as *Rlim*) and YY1/CTCF in the allelic regulation of *Xist* and *Tsix*^20–27^. The current model comprises a complex interaction network that is in a dynamic transcriptional equilibrium in undifferentiated ES cells^28^. In differentiating cells, *Xist* is strongly mono-allelically up-regulated, spreads onto the chromosome from which it is transcribed and silences genes *in cis*, including *Tsix* (reviewed in Van Bemmel et al. ^29^). It is known that the *Xist* promoter in undifferentiated ES cells is bivalent^30^, marked by both repressive and active histone modifications, and poised for activation. This state is resolved during differentiation at the up-regulated allele of *Xist*, which becomes actively transcribed and marked by a strong domain of H3K4me3^21,23^. In the context of these changes at the chromatin level in the vicinity of the *Xist* promoter, we hypothesise that chromatin remodelers may have a role in the onset of *Xist* regulation during cell differentiation.

ATP-dependent chromatin remodelers play essential roles in chromatin biology, compacting or relaxing the chromatin threads and, in turn, restricting or facilitating access of transcription factors (TFs), RNA polymerase II (Pol II) and the basic transcriptional machinery to the underlying DNA sequences^31^. In mammals there are about 30 different types of ATP-dependent chromatin remodelers, belonging to four major remodeler families^32^: SWI/SNF, ISWI, NURD-CHD, and INO80. Such complexes are implicated in most cell activities, from regulation of differentiation, to pluripotency and gene-specific activities. Chromatin remodelers can form hundreds of distinct functional entities by complexing amongst themselves in the cell, although the true extent of this phenomenon and the issue of tissue specificity, remain for the most part unexplored. There are however indications that these complexes have relatively little or no functional redundancy as single-gene Knock-Outs (KO) of chromatin remodelers generally produce observable and often very severe phenotypes^33^. For example, the BAF250 family, belonging to the SWI/SNF complex, is composed of three members (BAF250A–B–C). BAF250-A null embryos die at ~E6.5 and the defect is not compensated for by either BAF250B or BAF250C^34^. Similarly, *Chd2* mutants die before birth and other Chd-family members do not appear to compensate for such mutations^35^.

In order to focus our analysis on chromatin remodelers likely involved in the regulation of the initiation of XCI, we screened for candidate regulators, using the following criteria: (i) accumulation and binding to the X inactivation centre (XIC)^28,36^; (ii) robust expression during early mouse developmental stages^37,38^; (iii) a KO phenotype, where known, compatible with a role in XCI (MGI, www.informatics.jax.org/) or phenocopying XCI-defects. Based on these selection criteria and preliminary analysis, we decided to focus our immediate attention on the Chromodomain-Helicase-DNA-binding protein 8 (*Chd8*), which belongs to the CHD family. *Chd8* contains two chromodomains, a SWI/SNF2-like ATP-dependent helicase domain, several SANT/CR domains and two terminal BRK domains^31,39^. *Chd8* KO mice die in utero around E7.5–8 but appear to stop growing and start degenerating by E5.5, although sex-specific differences have not been reported^40^. A recent publication, which studied the role and the distribution of chromatin remodelers in ESCs, noted that different types of chromatin remodelers have specific histone substrate preferences, determining their genomic accumulation^36^. *Chd8* seems to accumulate in open-chromatin regions marked by H3K4me3 and DNA hyper-sensitivity sites^36^. *Chd8* has also been previously shown to regulate lncRNAs^41^ and autism-associated genes^42^. It is a key factor in correct neuronal differentiation, mutations which lead to autism spectrum disorders (ADS) phenotypes^41,43–45^. In our study, using undifferentiated and differentiating female ES cells and *Chd8* knock-down (KD) and KO systems, we demonstrate that *Chd8* has a critical role in *Xist* regulation. *Chd8* regulates strong yet controlled bursts of *Xist* expression, which are necessary for its correct spreading across the chromatin and initiation of XCI by preventing the spurious binding of TFs at the *Xist* promoter.

## Results

### Validation of experimental cell lines and conditions of differentiation

For our experiments we decided to use a previously developed and validated first-generation (F1) *129/castaneus* hybrid ES cell line carrying the insertion of a premature termination site in the *Tsix* gene (Fa2L)^46^. As a consequence of this mutation, when these cells are differentiated, the chromosome carrying the mutation (the *129* allele) is forced to become the inactive X chromosome (Xi) in ~95% of cells^46^. Furthermore, the extensive polymorphism between the *129* and the *castaneus* genomes facilitate allelic analysis. Single nucleotide polymorphisms (SNPs) between the strains are expected every 80–100 bp, on average^47^.

As female XX ESCs are known to be prone to lose one of their two X chromosomes, after extended passaging, we subcloned this line (Fa2L sub-clone 4; Fa2L-S4 hereafter) and verified the presence of two X chromosomes by DNA FISH (Supplementary Fig. 1c). In order to assess the efficiency of X chromosome inactivation initiation, we differentiated these cells from 2i conditions (undifferentiated state) to the neuronal progenitor cell state (NPCs) for 3 days (see the “Methods” section and below). We monitored the efficiency of XCI and differentiation by means of H3K27me3 Immunofluorescence (IF) staining, as a surrogate marker for the inactive X chromosome (Xi)^14,48^ and qRT-PCR analysis of selected differentiation (*Nestin*/*Rex1*) and XCI (*Xist/Tsix)* markers^17,19^ (see also the “Methods” section).

We show that after 3 days after differentiation induction, *Xist* and the NPC-specific marker Nestin are up-regulated and the pluripotency marker *Rex1* and *Tsix* (XCI marker) are correctly down-regulated. About 50% of the cells showed the presence of an inactive X chromosome by day 3 (H3K27me3 domains, Supplementary Fig. 1a, b). These data indicate that XCI has been robustly initiated by day 3 of differentiation, which was retained as the principal time point for subsequent analysis of *Xist* regulation during XCI initiation phase (see also Supplementary Fig. 4 and see the “Methods” section).

### Chd8 accumulates at the Xist promoter in undifferentiated and differentiated cells

**Fig. 1:**
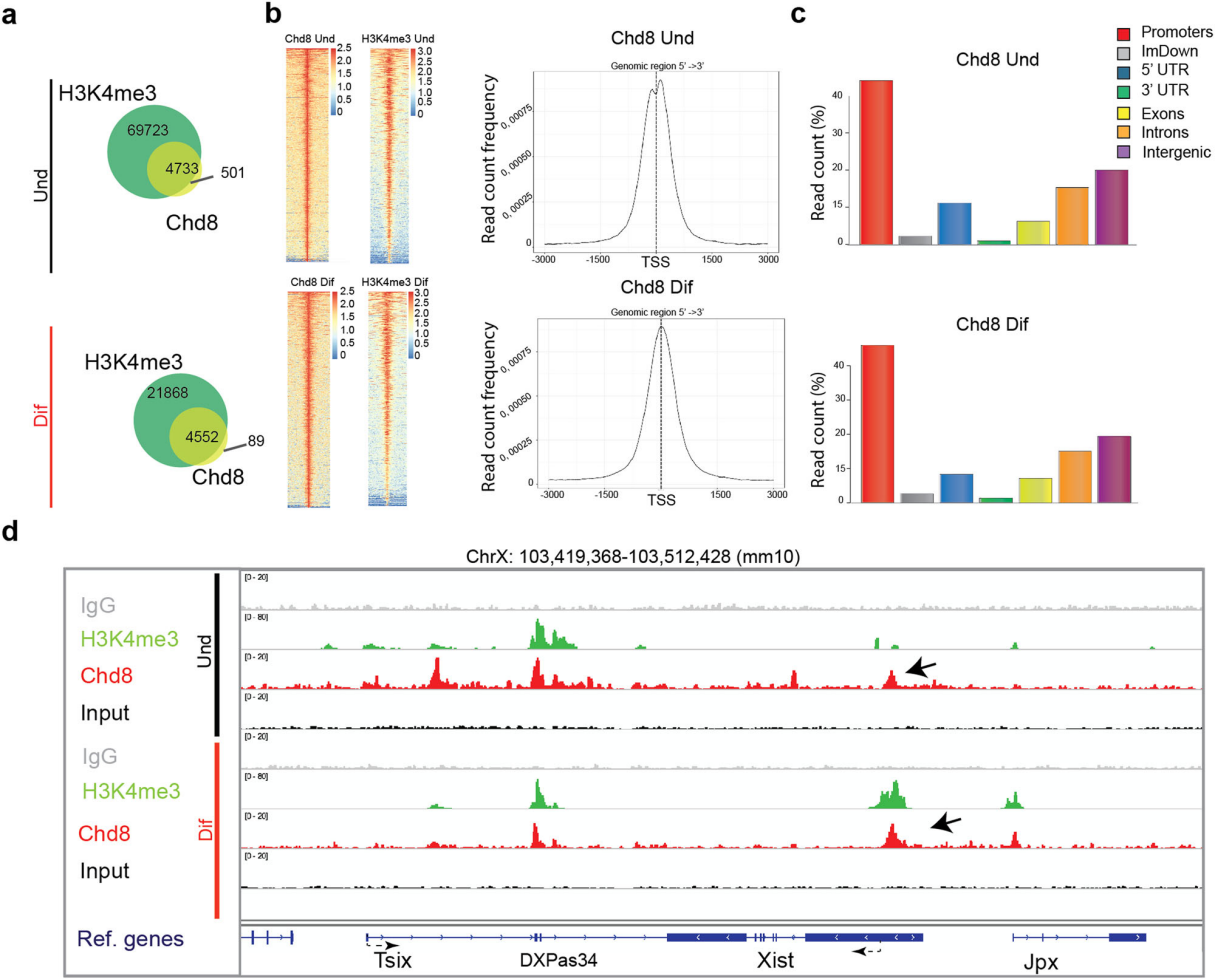
Characterization of the genomic distribution of *Chd8* and H3K4me3 peaks. **a** Overlap between *Chd8* and H3K4me3 peaks; top undifferentiated (Und) and bottom differentiated conditions (Dif) from two biological experiments. **b** Heatmaps showing the distribution of *Chd8* and H3K4me3 peaks in the genome (left). Association of *Chd8* peaks with transcriptional start sites (TSS); top undifferentiated, bottom differentiated conditions (right). Data from two biological replicas is shown. **c** Genomic features distribution of *Chd8* consensus peak sets; top undifferentiated, bottom differentiated conditions. Selected-features are shown (e.g. Promoters, Immediate downstream region (ImDown), etc.). Data from two biological experiments is shown. **d** *Chd8* distribution at aligned sequence reads at the *Xist* and *Tsix* promoter regions. Black arrows indicate *Chd8* peaks at the *Xist* promoter. A single representative set of ChIP-seq profiles is shown. Red: Chd8, green: H3K4me3, grey: IgG, black: input. Samples and antibodies used are indicated in the figure.

In order to determine the genomic distribution of CHD8 in our cell lines, we performed ChIP-seq experiments for CHD8 and H3K4me3, an active chromatin mark recognized by CHD8 chromo domains^30^. As expected, ~90% of CHD8 peaks overlap with H3K4me3 sites in undifferentiated conditions, and >95% in differentiating conditions (Fig. 1a). Genome-wide analysis of CHD8 peaks reveals that this protein localizes at transcriptional start sites (TSS), at promoters and at intergenic regions as previously reported^49^ (Fig. 1b, c and Supplementary Fig. 2). We went on to test whether *Chd8* and H3K4me3 are enriched at the X inactivation center (XIC) with a particular focus on the *Xist* or *Tsix* promoters^34^. We note—as expected—that during differentiation there is a shift in H3K4me3 accumulation from the *Tsix* regulatory region (major promoter and *DXPas34* tandem repeats) to the *Xist* promoter (Fig. 1d)^50^. We show that in ES cells (undifferentiated conditions) there is a CHD8 peak at the Xist promoter, whilst a slightly larger peak characterising this region under differentiating conditions (Fig. 1d). We reasoned that the CHD8 peaks might contribute to *Xist* regulation both in undifferentiated conditions and at the onset of XCI, potentially opening up the chromatin at the *Xist* promoter (see below).

Exploiting the SNP differences between *129* and *castaneus*, we performed allele-specific analysis of the H3K4me3 chromatin mark first, due to its abundance on chromatin, allowing for proper statistical analysis. We assigned ChIP-seq reads to the *129*, *castaneus* (Cast) or reference genome (BL6) (see the “Methods” section). As expected, during differentiation, most of the H3K4me3 is associated with the *129* allele of the *Xist* promoter (the future Xi) after immunoprecipitation but not in the pre-IP materials (input) (Supplementary Fig. 3a, b)^46^. CHD8 also appears to be mostly associated with the 129 allele, although due to the known limited number of reads from Chd8 ChIP-seq experiments^51,52^, we do not have the power to detect statistical differences between the alleles (Supplementary Fig. 3a, b). It is possible that this robust gain of H3K4me3, upon differentiation, is necessary for recruiting *Chd8* and/or other chromatin remodeler complexes to the *Xist* promoter through its chromodomains.

### Chd8 KD affects Xist up-regulation but it does not majorly affects XCI initiation

**Fig. 2:**
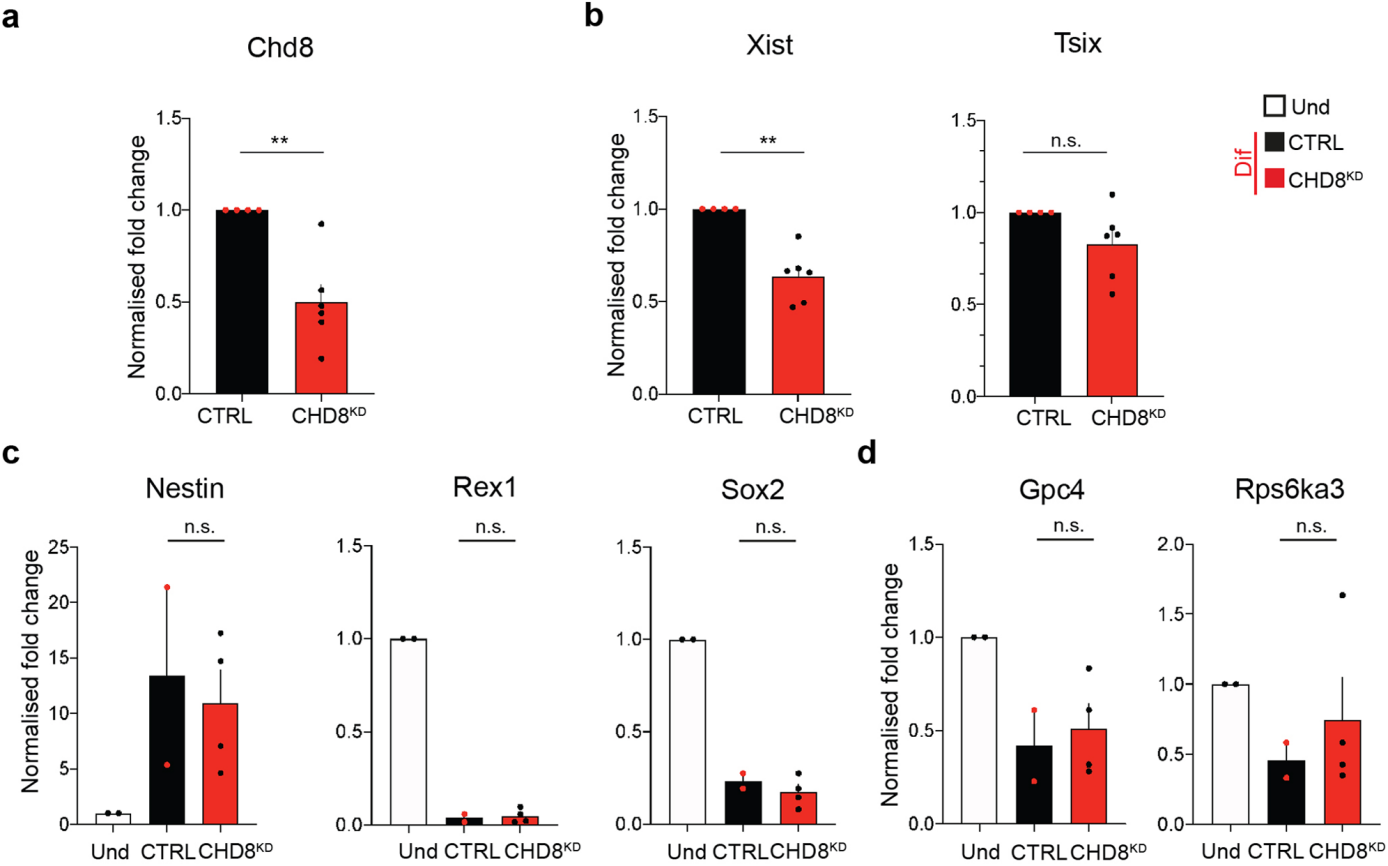
*Chd8* KD leads to *Xist* de-regulation. **a** CTRL-normalised *Chd8* qRT-PCR at 3-day differentiated cells in CTRL and *Chd8* KD cells is shown. CRTL: scrambled siRNA control (black bars), *Chd8*^KD^: specific siRNA pool to *Chd8* (red bars). Data from three independent experiments is shown. **b** CTRL-normalised *Xist* and *Tsix* qPCR at 3-day differentiated cells in CTRL and *Chd8*^KD^ cells is shown. CRTL: scrambled siRNA control (black bars), *Chd8*^KD^: specific siRNA pool to *Chd8* (red bars). Data from three independent experiments is shown. **c** Selected differentiation markers used for qRT-PCR analysis are shown. Data is normalised for the undifferentiated condition (Und). Data from two independent experiments is shown. **d** *Xist*-mediated gene silencing is not affected in *Chd8* KD (two randomly selected X-linked genes are showed). Data is normalised for the undifferentiated condition (Und). Data from two independent experiments is shown. Und (undifferentiated cells), Dif (3 day differentiated cells). Error bars represent standard error of the mean (SEM). Statistical significance was tested by means of two-tailed unpaired *t*-test (*p ≤ 0.05; **p ≤ 0.01). *p*-values, Xist = 0.00141, Chd8 = 0.00390. Single points represent independent biological samples. Gapdh was used as internal normalization control.

To analyse the functional role of *Chd8* in XCI initiation, we performed siRNA-mediated KD experiments in the parental cell line (Fa2L-S4). In order to choose the best time-point for siRNA delivery, we checked the dynamics of *Chd8* expression relative to that of *Xist* during differentiation. During differentiation, *Chd8* mRNA levels peak at day one and then decrease, concomitantly with an increase of Xist RNA (Supplementary Fig. 4a). Increases of *Chd8* at the protein level were detected slightly later, at day 2/3 of differentiation (Supplementary Fig. 4b, c). In view of the expression kinetics of these genes, we decided to KD *Chd8* at day 1 of differentiation, upon exit of the pluripotent state, when *Chd8* mRNA is at its maximum level, by means of siRNA (for 48 h). Using a pool of 4 siRNAs we efficiently knocked down *Chd8* at RNA level by ~50–60% at day 3 of differentiation (Fig. 2a). The progression of XCI and differentiation was analyzed by means of qRT-PCR and IF (see also “Methods” section) (Fig. 2 and see the “Methods” section). We noted that this partial *Chd8* KD led to significantly reduced *Xist* expression (~30%) during differentiation (Fig. 2a), while *Tsix* down-regulation, cell differentiation markers (Rex1, Sox2, Nestin) and *Xist*-mediated gene-silencing processes were unaffected (Fig. 2b, c). We assessed whether the reduced *Xist* expression under *Chd8* KD conditions during differentiation is associated with defective XCI initiation. XCI initiation was followed in differentiated control and *Chd8* KD cells by means of H3K27me3 IF. Initiation of XCI was slightly reduced at day 3 of differentiation in KD vs. WT cells (Supplementary Fig. 5). As the H3K27me3 domains in *Chd8* KD are indistinguishable from the parental line in size (Supplementary Fig. 5), this observation suggests that potentially fewer cells may initiate XCI in the *Chd8* KD-treated cells. RNA-seq data, shows in *Chd8* siRNA vs. Control siRNA (Diff condition), 11 differentially expressed genes (DEG, false discovery rate (FDR) ≤ 0.05, and absolute log2FC ≥ 1—Supplementary Data 1), of which 6 were upregulated and 5 downregulated.

This relatively minor variation in gene expression suggests that the differentiation process is not, per se, affected by partial *Chd8* KD treatment (Supplementary Fig. 6a). Principal component analysis (PCA) reveals that the overall expression patterns of the *Chd8* KD cell line, during differentiation, is very similar to that of the control cell line (Supplementary Fig. 6a). RNA-seq analysis confirmed *Chd8* down-regulation (≥50%) was accompanied by only a small and statistically non-significant reduction in *Xist* expression (~17%). Differences in sensitivity and substrate preference between the qRT-PCR and the RNA-seq data likely explain such low-level discrepancies. Indeed, while qRT-PCR can capture both poly-Adenylated (PolyA+) and non-poly-Adenylated RNAs (PolyA−), our RNA-seq experiments were specifically designed to capture exclusively the cellular PolyA+ RNA pool (see the “Methods” section). Considering that a large proportion of *Xist* RNA is not polyadenylated^53,54^, these results are in line with our expectations. For these reasons, from this point, we will only refer to qRT-PCR analysis for *Xist* and *Tsix* paired quantification.

In order to assess the extent of *Xist* dependency on the levels of CHD8, we also generated two *Chd8* KD stable cell lines by lentiviral transduction showing respectively mild and severe *Chd8* KD (Chd8.1^KD^, Chd8.2^KD^, Chd8.1/Chd8.2 hereafter in the text) (Supplementary Fig. 7a, b, see also the “Methods”). In cell differentiation experiments, we noted that the mild *Chd8* KD (Chd8.1), in agreement with the siRNA-mediated *Chd8* KD, is associated with a reduced upregulation of *Xist* upon differentiation by qRT-PCR, although in this case, this variation was not statistically significant (*p* = 0.082). The discrepancies between the siRNA mediated and light shRNA *Chd8* KD line (Chd8.1), might be due to differences in level of KD achieved in the siRNA vs the shRNA experiment (50–60% vs. ~40% RNA KD at the RNA level, respectively); or to the experimental set up (such as stable vs. transient *Chd8* KD, see the “Methods” section). In the Chd8.1 cell line, qRT-PCR analysis, showed cell differentiation was not affected in this cell line at 3 days of differentiation in this cell line, using established markers (Supplementary Fig. 7c). We went on to test the role of mild CHD8 depletion during the XCI initiation phase by means of H3K27me3 IF in these stable KD cell lines. In the *Chd8*.1 cell line, we showed a modest reduction in the number of cells that initiate XCI (Supplementary Fig. 7a), similar to that seen for the siRNA-mediated KD (Supplementary Figs. 2 and  5). In the severe CHD8 KD (Chd8.2 ≥ 80% Chd8 KD), we show a modest but significant increase in Xist expression and a concomitant increase of *Tsix* repression, at the onset of XCI. Overall cell differentiation was not significantly affected, and no change in the number of cells initiating XCI at 3 days of differentiation was observed using H3K27me3 as marker of the Xi (Supplementary Fig. 7a, c).

RNA-seq analysis of these *Chd8* KD lines (Supplementary Fig. 8, Supplementary Data 2), showed no major defects in cell differentiation and only a minor number of genes significantly deregulated for the Chd8.1 line (35 significantly deregulated genes, 8 of which upregulated and 27 downregulated; FDR ≤ 0.05, log2FC ≥ 1). RNA-seq analysis for the Chd8.2 line revealed more significantly deregulated genes, as expected from the higher level of CHD8 KD efficiency. We report 213 DEG in Chd8.2, roughly equally divided in upregulated and downregulated genes (108 downregulated and 105 upregulated; FDR ≤ 0.05, absolute log2FC ≥ 1).

These unexpected differences between light (siRNA/Chd8.1) and severe Chd8 KD (Chd8.2) (Supplementary Figs. 5 and 7) suggests the existence of a threshold effect in *Chd8*-dependent *Xist* regulation during XCI (see also Discussion).

### Chd8 KO affects Xist regulation and XCI initiation

**Fig. 3:**
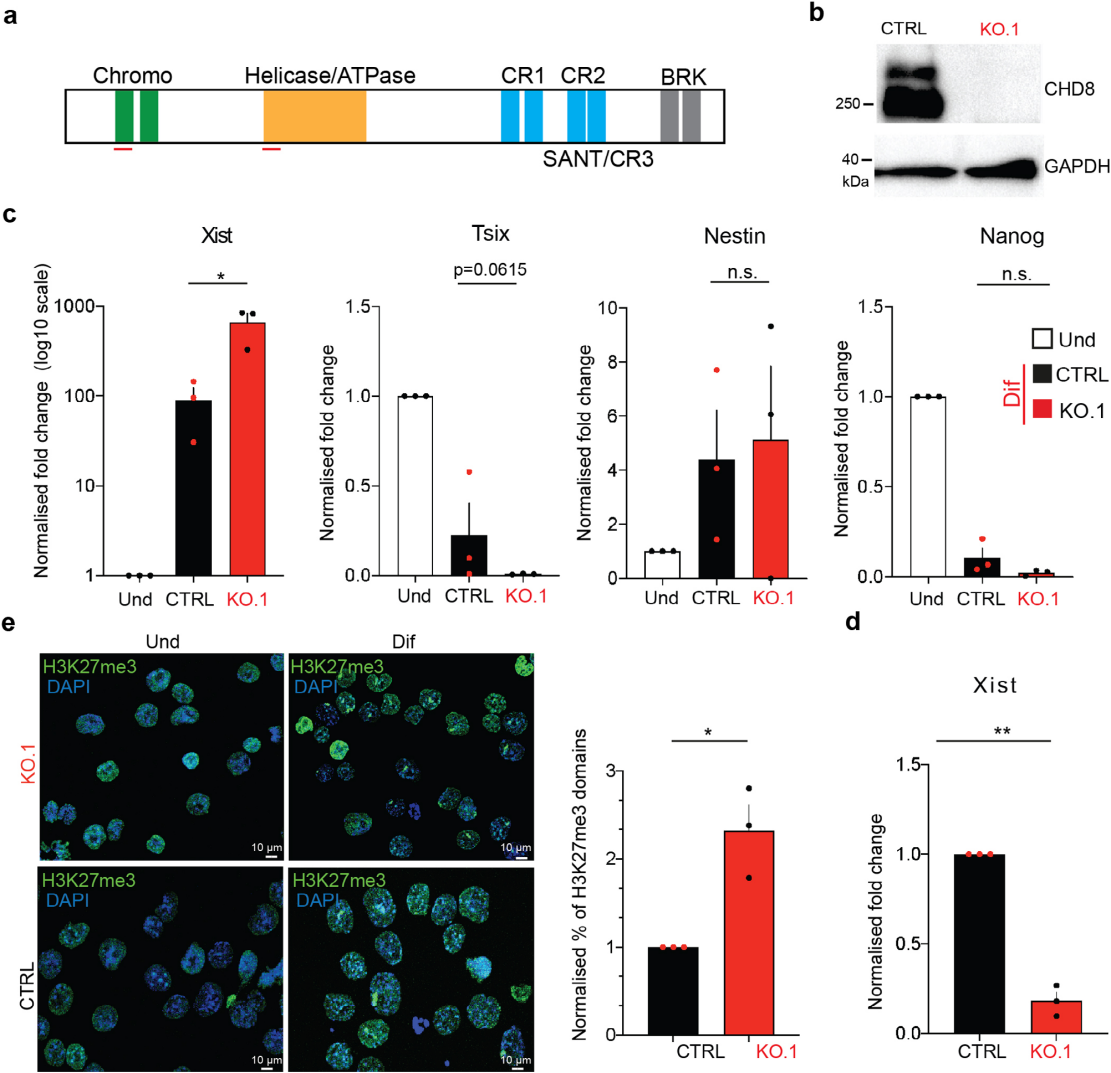
*Chd8* KO affects *XCI* initiation. **a** Schematic representation of *Chd8* protein domains; red lines indicate the position of the CRISPR/Cas9 guides used (not in scale). **b** Western Blot analysis of *Chd8* KO.1 (clone C4.1) and parental ES cells (CTRL, Fa2L-S4) are shown. **c** qRT-PCR results showing expression levels of *Xist, Tsix*, *Nestin*, *Sox2*. In differentiating ESCs *Xist* is strongly up-regulated in *Chd8* KO cells whilst cell differentiation is not affected (*Nestin*, Nanog); CTRL, parental cell line: Fa2L-S4; KO, sub-clone C4.1. Data is normalised for the undifferentiated condition (Und). Data from three independent experiments is shown. *p*-values, Xist = 0.0367. **d** *Xist* qRT-PCR data from parental (CTRL) and KO.1 cells in undifferentiated state is shown. Und (undifferentiated cells), Dif (3 day differentiated cells). Data from three independent experiments is shown. Error bars represent standard error of the mean (SEM). Statistical significance was tested by means of two-tailed unpaired *t*-test (*p ≤ 0.05; **p ≤ 0.01). *p*-values, Xist = 0.00362. Single points represent independent biological sample. Gapdh was used as internal normalization control. **e** Representative images of H3K27me3 in CTRL vs. *Chd8* KO cells (left) and the normalised scoring of H3K27me3 domains (IF) is shown (right), *n* = 658. Data from three independent experiments is shown. Error bars represent standard error of the mean (SEM). Statistical significance was tested by means of two-tailed unpaired *t*-test (*p ≤ 0.05; **p ≤ 0.01). CTRL vs. KO.1, *p*-value = 0.0463. Single points represent independent biological samples.

In our KD experiments, we achieved up to ~80% *Chd8* RNA/protein KD efficiency, which would likely still leave a reasonable amount of protein available in the cell. In order to ablate the protein completely, we used CRISPR/Cas9-mediated gene editing. We designed guides targeting the chromo-domains and helicase-domains of *Chd8*, with the aim of inducing a frame-shift mutation upon CRISPR/Cas9-mediated non-homologous end-joining (NHEJ) repair (Fig. 3a, and see the “Methods” section). We obtained several clones carrying mutations in either or both domains. We decided to proceed with two sub-clones (C4.1 and C4.3; KO.1 and KO.2 hereafter; see the “Methods” section) carrying a 20 bp deletion/1 bp insertion in the helicase domain and 5 bp deletion in chromo domain (see the “Methods” section). Both mutations are predicted to generate frame-shift mutations. The absence of *Chd8* protein was verified by sequencing and Western Blot (WB) analysis, respectively (see the “Methods” section) (Fig. 3b).

Expression of *Xist* in differentiated cells by qRT-PCR—normalised over the undifferentiated state — was revealed to be increased rather than decreased, mimicking, in part, the results seen with the severe *Chd8* KD (Chd8.2). XCI initiation was revealed to be enhanced by up to ~1.5/2 fold by H3K27me3 IF analysis (Fig. 3e). The differentiation process per se using established markers, does not appear to be critically affected (Fig. 3c). We also report that undifferentiated C4.1 cells show a significant reduction in *Xist* basal expression (Fig. 3d). Equivalent results were found in the other *Chd8* KO clone (KO.2).

RNA-seq analysis of the *Chd8* KO lines over the parental line (Fa2L-S4), shows more widespread gene deregulation compared with the *Chd8* si/shRNA KD (Supplementary Fig. 12). In particular, we report 2485 DEG genes, 857 of which are upregulated and 1628 downregulated (FDR ≤ 0.05, absolute log2FC ≥ 1, Supplementary Data 3). Noticeable, 160/213 (75.1%) deregulated genes from the severe *Chd8* KD (Chd8.2) overlaps with the *Chd8* KO-deregulated genes. The differentiation process, per se does not appear to be markedly compromised (see the “Discussion” section).

### Chromatin accessibility at the XIC

**Fig. 4:**
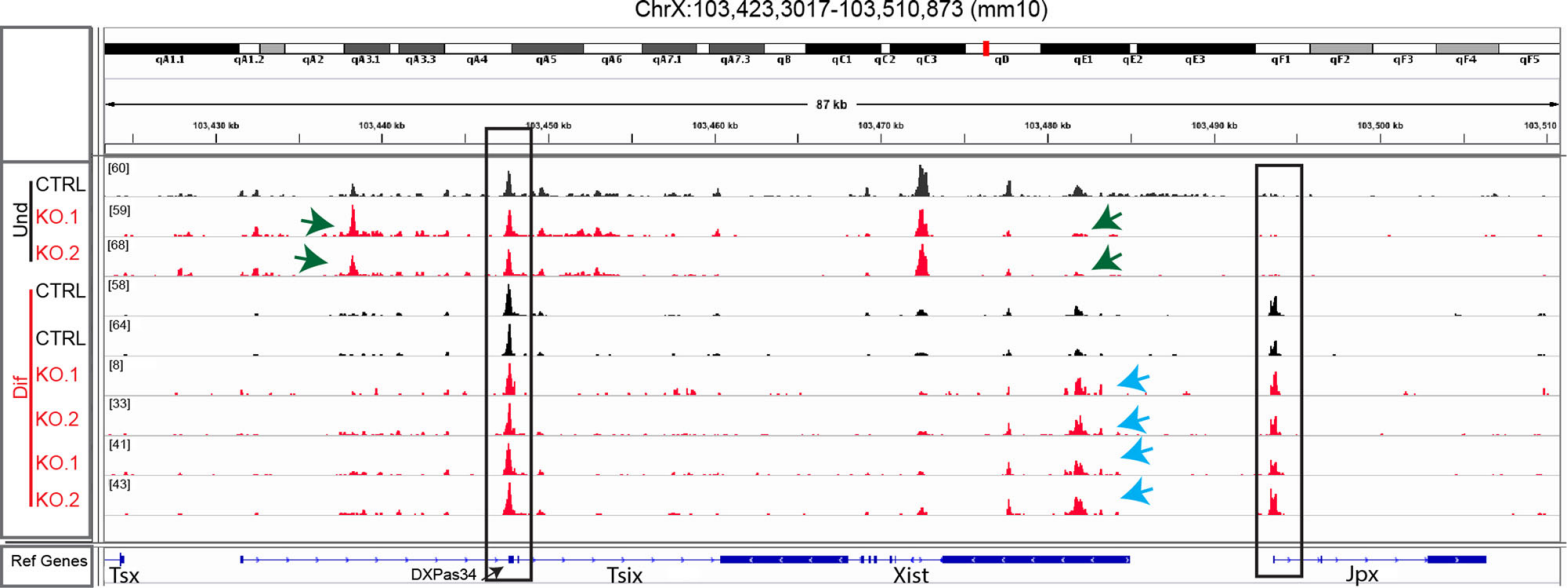
ATAC-seq analysis zoom at the *Xist* locus. Zoom-in on the XIC around the *Xist* and *Tsix* genes. In black the parental cell line and in red two representative KO cell lines (C4.1/C4.3 sub-clones). Undifferentiated and differentiating ES cells are shown (Und/Dif). Light-blue arrows indicate changes in chromatin structure at the *Xist* promoter in differentiating cells. A black box surrounds the *Tsix* and *Jpx* main regulatory regions, indicates no change of chromatin structure. Green arrows indicate chromatin changes around the *Tsix* promoter/*Tsix* intron and *Xist* promoter, (CTRL, parental cell line: Fa2L-S4; *Chd8* KO.1, sub-clone C4.1; *Chd8* KO.2, sub-clone C4.3).

We decided to assess whether *Chd8* KO could affect local chromatin organization of the XIC, with a particular focus on the *Xist* and *Tsix* regulatory regions. To this end we performed ATAC-seq analysis on undifferentiated vs. differentiated *Chd8* KO cells in clone C4.1 (KO.1) and C4.3 (KO.2) (Fig. 4). Whilst under undifferentiated conditions we saw no changes in chromatin accessibility at the *Tsix* major regulatory regions (e.g. Ex2/3 and *DXPas34* microsatellite) nor at the Jpx promoter, we observed an increased accessibility at *Tsix* intron one and a small reduction in chromatin accessibility at the Xist promoter. Under differentiating conditions, we noted a marked increase of chromatin accessibility at the *Xist* promoter (Fig. 4). These results are in good agreement with the qRT-PCR and H3K27me3 IF data shown in Fig. 3.

### Complementation assay

**Fig. 5:**
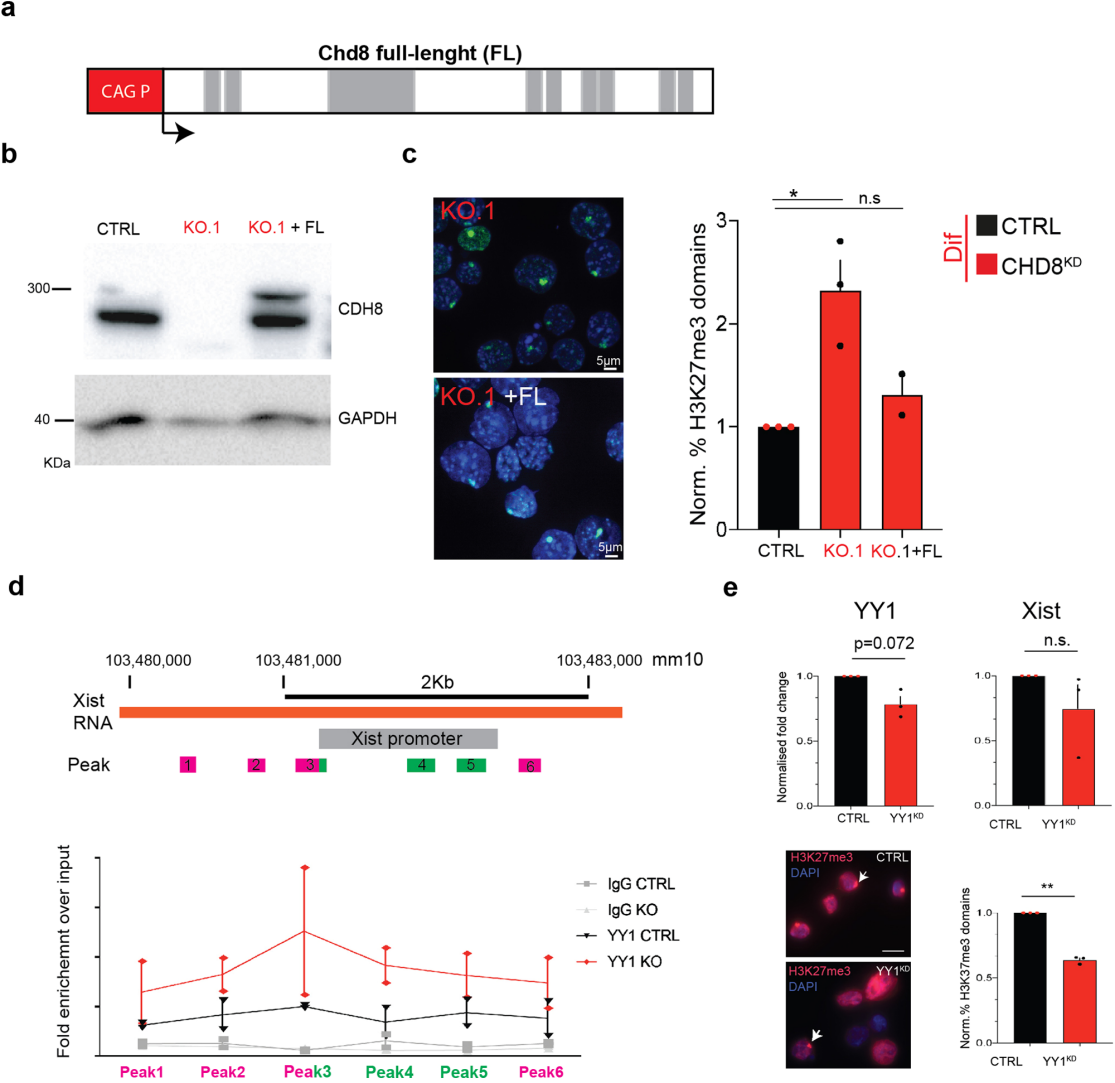
Complementation of Full-length (FL) *Chd8* into *Chd8* KO cells. **a** Schematic representation of the *Chd8* expression construct. Protein domains are indicated. CAG promoter is shown (CAG P) in the red box. **b** Western Blot analysis of the *Chd8* KO line (C4.1) and complemented cells. Top CDH8 and bottom loading control (GAPDH) blots are shown. **c** H3K27me3 IF analysis of *Chd8* KO and complemented cells. Right representative images, left CTRL-normalized quantification of the number of cells having an Xi at 3 days of differentiation. To test for statistical significance, our data was fitted using Poisson regression method and multiple correction testing (*p ≤ 0.05). Data from two and three experiments are shown, *n* = 827. Single points represent independent biological samples. Standard error of the mean (SEM) is shown. **d** Top, schematic of *Xist* promoter and primers used for analysis (peaks 1–6). Bottom, qPCR analysis of YY1 Cut&Run qPCR analysis at the *Xist* promoter. Data from two experiments are shown. Single points represent independent biological samples. Matched input samples were used as normalization control. Sample names are shown in the legend. **e** Top, CTRL-normalised qRT-PCR analysis of scrambled siRNA (CTRL) and YY1 siRNA (YY1^KD^) in Chd8 KO.1 (KO.1 line). Tested genes are indicated. Statistical significance was tested by means of two-tailed unpaired *t*-test (*p ≤ 0.05). Data from three experiments are shown. Bottom, representative images of H3K27me3 in CTRL vs. YY1^KD^ cells (left) and the CTRL-normalised scoring of H3K27me3 domains (IF) is shown (right), *n* = 744. White arrows indicate examples of H3K27me3 domains. Statistical significance was tested by means of two-tailed unpaired *t*-test (*p ≤ 0.05). CTRL vs. YY1^KD^, *p*-value = 0.00196. Data from three experiments is shown. Gapdh was used as internal normalization control. Single points represent independent biological samples.

In order to exclude potential cell-line specific artefacts, we decided to transfect the KO cell line (C4.1) with a plasmid expressing the full-length (FL) CHD8 protein. To this end, we generated, by bacterial recombineering, a FL *Chd8* protein under the control of a constitutive CAG promoter (Fig. 5 and see the “Methods” section). Reconstituted CHD8 protein levels appear to be comparable with the parental cell line by WB analysis, a necessary condition for our analysis (Fig. 5). In brief, complemented cells were differentiated and XCI progression was assessed by H3K27me3 IF. As expected the FL form of CHD8 effectively rescued the defective KO phenotype, in the context of XCI initiation (see the “Methods” section and Fig. 5b, c). This indicates our results are unlikely to be majorly influenced by line-specific factors (see the “Discussion” and the “Methods” section), but rather linked to *Chd8*-specific regulation.

### Candidate and unbiased TFs binding analysis at the Xist promoter and Chd8 mass-spectrometry

Taking into consideration the results obtained in *Chd8* KD and KO conditions, we hypothesised a competitive binding at the *Xist* promoter between TFs and CHD8. In brief, one might expect that some factors would increase (or decrease) their binding at the *Xist* promoter, in the absence of CHD8, and contribute to the observed transcriptional deregulation that we observed, in *Chd8* KO lines.

In order to determine whether in the absence of CHD8, another transcription factor binds at the *Xist* promoter in a non-physiological fashion (i.e. increase or decrease), we decided to use two complementary approaches. First, using known databases of ChIP-seq (https://chip-atlas.org/, see also the “Methods” section), we screened for all factors binding at the *Xist* promoter and expressed in ES and differentiating/differentiated conditions. On this basis, we selected YY1 and INO80 for further analysis, and excluded *Nanog* and *Rex1* as they are not expressed in differentiated cells, are severely reduced in differentiating cells, and are known *Xist* repressors^29^. We also excluded from our analysis CTCF as its reduction during differentiation does not affect proper XCI initiation^55^. We also used a motif analysis approach (MEME, see the “Methods” section) in order to reveal, in an unbiased way, potential TFs competing with CHD8 at the *Xist* promoter. To this end, we extracted all the sequences underlying our CHD8 peaks (ChIP-seq analysis) and used these as bait to find potential consensus motifs shared with other TFs. The analysis revealed the enrichment of several known TF-binding motifs, including Egr1, SP1, SDPEF, and YY1 (Supplementary Fig. 9a), In particular, YY1 attracted our attention for its known role as a major *Xist* activator^56^, and we decided to further test this candidate (see below).

Finally, in order to have an unbiased candidate approach as well, we performed CHD8-IP followed by mass spectrometry analysis. We decided to do both native protein IP using two antibodies toward CHD8 and a tag-mediated (HA-tag) IP in order to recover only the strongest candidate. To this end, we decided to generate a *Chd8* KI cell line carrying a C-term tag (3Flag-HA-TAG; 3FHT in short) (Fig. S10) to be used for the HA-mediated IP (for details about the generation and validation of this cell line, please refer to the “Methods” section). Interestingly, CHD8-mediated and HA-mediated IPs followed by mass spectrometry, revealed WDR5, a known CHD8-interacting protein both in mouse and human (STRING, see the “Methods” section) as main CHD8 partner (Supplementary Data 4 and Supplementary Fig. 9c). As WDR5 is a known component of the MLL complex and a strong activator of gene transcription and a Polycomb-repression antagonist^57^, we decided to include it in our analysis.

We tested these three factors by ChIP-seq or ChIP/Cut&Run qPCR analysis. In brief, by ChIP-seq analysis, we could not detect any change in INO80 occupancy at the *Xist* promoter and neighbouring regions, in the absence of CHD8. Hence, we excluded this factor as a potential candidate competing for the *Xist* promoter (Supplementary Fig. 11). Similarly, using Cut&Run qPCR analysis, we could not detect any enrichment of WDR5 in differentiating conditions, either in the parental WT line nor for the *Chd8* KO line, at the *Xist* promoter. This suggests that it is unlikely that WDR5 is playing a major role in *Xist* upregulation during XCI initiation (Supplementary Fig. 11b). On the other hand, when we performed Cut&Run analysis for YY1, and we found an increase of this transcription factor at the *Xist* promoter (peaks 3–4, Fig. 5d), overlapping with the CHD8 peaks. Because YY1 is a known (strong) activator of *Xist*^56^, we reasoned that the elevated binding of YY1 at the *Xist* promoter, is very likely to be responsible of abnormal activation of *Xist* in the absence of *Chd8* protein (see also below). In order to test YY1’s role in *Xist* transcription in the absence of *Chd8*, we decided to perform YY1 KD during differentiation in CHD8-depleted cells (Chd8 KO.1). As YY1 KD is known to affect cell/neuronal differentiation^58,59^, we decided to perform mild siRNA-mediated YY1 KDs (i.e. 20–30% reduction), not to affect overall cell differentiation (Fig. 5e, Supplementary Fig. 11c). qRT-PCR analysis of YY1 KD lines over control, revealed a minor reduction of *Xist* RNA (Fig. 5e). This reduction is, however, linked to a significant reduction of cells initiating XCI as previously reported^56^, but not to the abolishment of XCI initiation (Fig. 5e). These observations indicate that other factors (i.e. redundancy) are likely involved in the upregulation of *Xist*, other than *Chd8* and YY1^60,61^.

## Discussion

Whilst many aspects of *Xist* regulation have been intensely studied over the years^17–24,62^, little is known about the role of chromatin remodelers in the context of XCI. Here we show that *Chd8* is a likely critical factor involved in the regulation of *Xist* expression.

**Fig. 6:**
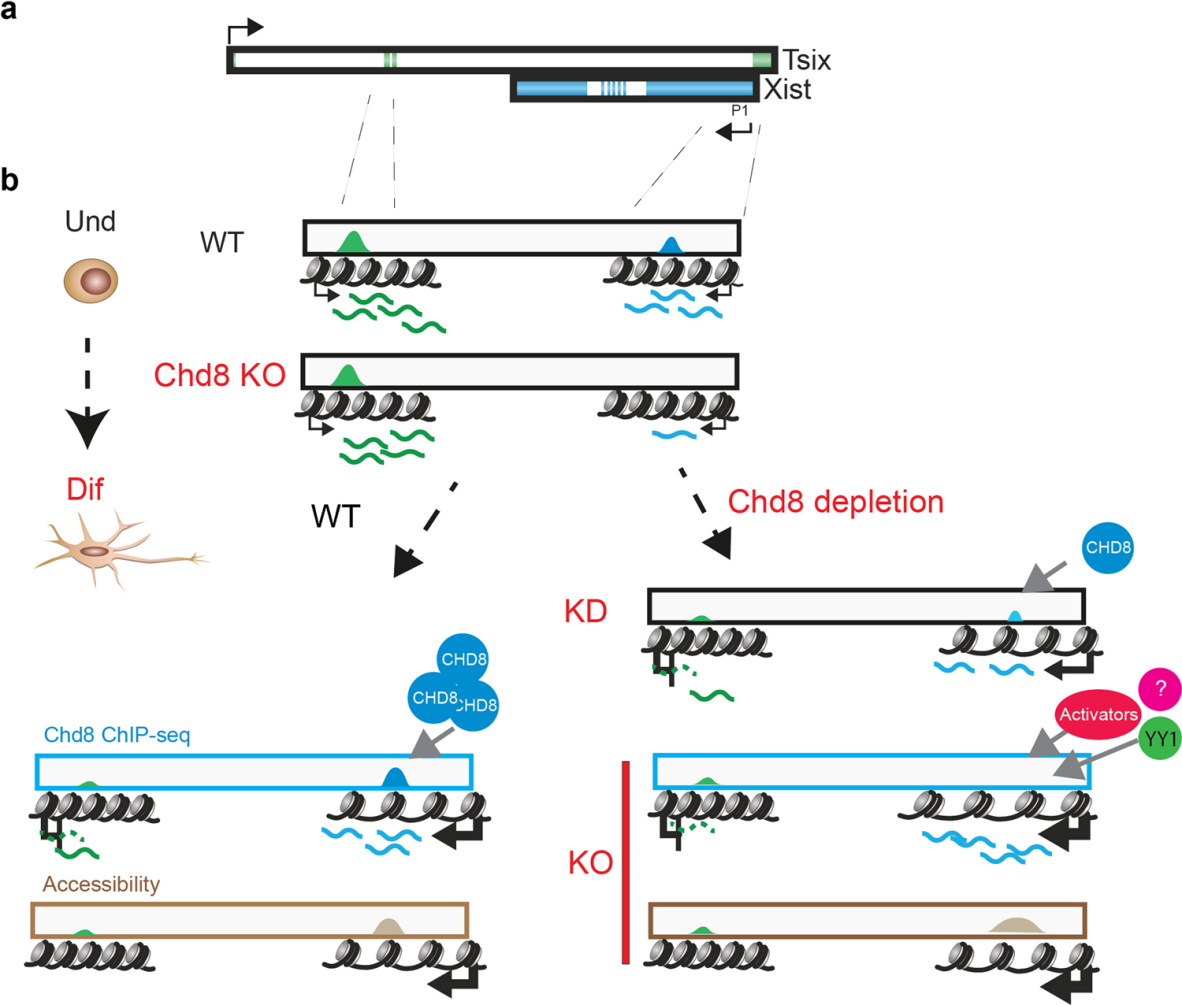
Proposed model of action of *Chd8* on the *Xist* promoter. **a** Schematic representation of the *Xist* (blue) and *Tsix* genes (green). **b** *Chd8* is crucial for correct *Xist* expression in undifferentiated and differentiating ESCs. CHD8 is shown in light-blue; *Tsix*-specific TF/remodelers are shown in green. Top, CHD8 localization at the *Xist* promoter is shown (light-blue peak). In undifferentiated conditions (Und), in the absence of CHD8, there is reduction of the basal transcription level of *Xist* (blue wavy lines), while *Tsix* transcription level is unaffected (green wavy lines). **c** Differentiating conditions in *Chd8* KD and *Chd8* KO background. Right, a mild *Chd8* KD leads to a small reduction of *Xist* steady levels (KD). Complete and severe CHD8 depletions instead, lead to increased *Xist* expression and a further *Tsix* downregulation (blue wavy lines *Xist*, green wavy lines *Tsix*). This condition also leads to a more-open chromatin state of the *Xist* regulatory regions, but not at *Tsix*’s, during differentiation (light-brown peaks). We suggest that YY1 (green circle) and/or known (red circle) and yet-to-be-identified protein (purple circle with question mark) may bind to the *Xist* promoter in the absence of the CHD8 protein (red arrow) and strongly activate it. Left, WT situation is depicted.

Our data suggest the involvement of *Chd8* as an activator of *Xist* in undifferentiated ES cells (Figs. 3 and 4) and in the fine-tuning *Xist* expression during differentiation (Figs. 1–4). We show by *Chd8* KD experiments (Fig. 2, Supplementary Fig. 7), that correct *Xist* transcriptional activation appears to depend on the presence of WT-levels of *Chd8* protein, recruited specifically to the *Xist* promoter via recognition of an H3K4me3 mark, which is specifically enriched on the allele chosen for X inactivation at the very onset of differentiation (Fig. 1 and Supplementary Fig. 3). In differentiating cells, *Chd8* subsequently fine-tunes the amplitude of *Xist* expression (Figs. 3–5), we hypothesise with CHD8 binding at the *Xist* promoter, restricting alternative binding of transcriptional co-activators to its regulatory regions, via a competitive binding exclusion. This implies that in the absence of CHD8, co-activators and other TFs could bind to the *Xist* promoter and lead to transcriptional up-regulation (Fig. 6). In this context, known regulators of *Xist* transcription, such as YY1, might bind to the *Xist* promoter with greater avidity or more robustly. YY1 has been previously shown to bind to XCI escapees, including the *Xist* promoter^63^ and to be a strong activator of *Xist* transcription, competing with REX1 for binding at the *Xist* promoter^56^. Our analysis suggests that YY1 is one of the most-likely activators of *Xist* transcription in the absence of *Chd8* protein since we observed a marked increase of YY1 recruitment at the *Xist* promoter in the absence of CHD8 (Fig. 5d, Supplementary Fig. 9). Indeed, our analysis of YY1 KD in *Chd8* KO cells confirmed YY1’s role in the initiation of XCI and *Xist* regulation (Fig. 5e). YY1 KD in *Chd8* null background, also supports the possibility that other factors are involved in *Xist* regulation^61^ (i.e. the absence of key regulatory proteins can be compensated by other proteins). The comparison of publicly available YY1 ChIP-seq datasets^64^ with our *Chd8* ChIP-seq data in embryonic stem cells revealed ~75% of overlap between YY1 and *Chd8* peaks. Furthermore, of the 2484 DFG in the *Chd8* knockout (differentiated), 1203 promoters are bound by *Chd8* and of those 391 also by YY1 (see the “Methods” section). This further suggests competition or cooperative effects in the regulation of target genes.

Other candidates competing for the *Xist* promoter that were tested in this study (INO80 and WDR5), did not show any occupancy change at the *Xist* promoter in *Chd8* KO vs. WT conditions. These factors, therefore, are unlikely to play an important role at *Xist* promoter, under the tested conditions. Our experiments using different *Chd8* KD lines, also suggest that CHD8 levels have a threshold effect on *Xist* regulation. Mild *Chd8* KD downregulates *Xist* expression, supporting its role as a transcriptional activator^65^. Conversely, strong CHD8 depletion (KO) has the opposite effect, most likely through competition with TFs^56^ as discussed above.

Whilst our data fully supports a role for *Chd8* as a transcriptional activator of gene-expression as previously shown^49,65^, *Chd8* might also act as a repressor of transcription^66^. One possibility is that *Chd8* regulates *Xist* expression under differentiating conditions by recruiting the histone H1, to moderate the amplitude of *Xist* up-regulation^66^. While our experiments cannot fully discriminate between the activating and/or repressive activities of *Chd8*, our data, and in particular the ATAC-seq experiments strongly support an activating role for CHD8 rather than a repressive role in *Xist* regulation. A possible complication in the comparison of WT vs. KO cells is that the cell differentiation in *Chd8* KO cells could have a different differentiation kinetic from that of the matched parental cell line, with more differentiated cells at day 3, in line with previous reports^44,52,67^. We did not find any convincing evidence of such neuronal differentiation enhancement in *Chd8* KO by RNA-seq analysis^49,52^ (Supplementary Fig. 12a, b). Finally, RNA-seq analysis of all the XIC factors^28^ in *Chd8* null background (vs. control), indicates that *Rnf12*, *Ftx*, *Xist* main activators, and Chic1 are significantly downregulated (Supplementary Data 3). This further suggests that *Xist* upregulation is unlikely to be linked to an indirect effect mediated by these factors.

In summary, our experiments support the hypothesis that *Chd8* fine-tunes *Xist* expression amplitude levels and regulates occupancy at the *Xist* promoter. In the absence of CHD8 other protein(s) are allowed to bind (or not to bind) at the *Xist* promoter inducing concomitant deregulation (Fig. 6). Our results have a broader applicability for the interpretation of the phenotypes observed in KO animals or cells. For example, the KO of a gene known to repress a given cadre of target genes, might result in the opposite effect (i.e. upregulation) in the case other TFs, such as an activator, is now allowed to engage with these regulatory sequences. Finally, our study emphasises our relative ignorance of the nature and importance of the interactions occurring between XCI regulation and chromatin remodeler activity suggesting the interest of additional studies on other remodelers in *Xist* regulation.

## Methods

### Construct generation

We purchased the FL *Chd8* cDNA from Genescript and cloned it into the MreI and NotI sites of pCAGp292-Puro. The MreI-NotI *Chd8*-containing fragment from this plasmid was cloned into the same sites of the plasmid, pCAG-mCherry-modified (this work), leaving the CAG promoter intact to create pCAG-*Chd8*-Neo. This plasmid was cut with PvuI, then blunted, cut with EcoRV and re-ligated to create pCAG-*Chd8*-Neo-AmpS. To aid in identifying the correct recombinant plasmids, bacterial strains that produce Cas9 and a sgRNA against the sequence in question were made by recombineering. Sequencing confirmed that surviving plasmids contained the precisely designed deletion. Plasmid maps and sequences are available upon request.

### KI generation and sub-cloning

*Chd8* KI cell lines were generated by standard DNA-recombineering. About 20 µg of NotI-linearized plasmid was electroporated into the Fa2L recipient cell line, an F1 *129/castaneus* hybrid ES cell line carrying the insertion of a premature termination site in the *Tsix* gene (Fa2L)^46^. Two days after transfection, cells were selected with G418 at a concentration of 250 µg/ml for ~7 days followed by Gancyclovir at 2 µM concentration for 2–4 days. After 10–11 days, resistant colonies were picked and expanded. Using homologous recombination, we obtained several 3′ KI cell lines and selected clone 3B for further analysis (we will refer to this clonal cell line as *Chd8*KI hereafter) (Supplementary Fig. 10). We showed by Southern Blot (SB) and long-range PCR (LR-PCR) analyses the correct integration of our construct (Supplementary Fig. 10a, b). We verified, in this newly generated cell line, the presence of randomly selected *129/*castaneus SNPs by Sanger sequencing (Supplementary Fig. 10c), the presence of two X-chromosomes by DNA FISH (Supplementary Fig. 10d), and the expression of the tagged allele by WB analysis (Supplementary Fig. 10e).

### Characterization of differentiation of the Chd8^KI^ line

In order to assess the efficiency of X chromosome inactivation initiation we differentiated the *Chd8*KI clone from 2i conditions to the NPCs for 3 days (see below). We monitored the progression of XCI and differentiation by H3K27me3 IF staining, as a surrogate marker for the inactive X chromosome (Xi) and used qRT-PCR for analysis of differentiation (*Nestin/Rex1*) and XCI (*Xist/Tsix*) markers.

*Xist* and NPC-specific Nestin were found to be correctly up-regulated, and the pluripotency marker Rex1 and *Tsix* properly down-regulated during differentiation with some 50–60% of the cells showing an inactive X chromosome by day 3 of differentiation (Supplementary Fig. 10f, g). These data indicate that XCI was robustly initiated by day 3 of differentiation.

We also sub-cloned the parental cell line (Fa2L) in order to obtain a homogeneous line of cells carrying two X chromosomes (Fa2L sub-clone 4; Fa2L-S4). Sub-cloning was achieved by plating cells at very-low density (500 cells/plate) in 10 cm tissue culture dishes then waiting 7–9 days before picking individual colonies and re-plating these in 2i medium Single colonies were expanded and characterised using routine procedures. The Fa2L-S4 line was characterized as described above for the *Chd8* KI line (see also main text for details).

### Cell culture conditions

Cells were grown in 2i conditions as previously published^68^. For differentiation experiments, the wells of a six-well plate or a 10 cm TC dish were pre-coated with gelatin (0.1%) containing laminin (1:1000) solution and incubated at 37 °C for 15 min. 150,000 cells per well in six-well plate (or 0.8 million for 10 cm dish) were plated in each well in neuronal differentiation medium (NDM) as previously published^69^. The cells were differentiated for 3–4 days and harvested for ChIP-seq, qRT-PCR work or IF/RNA-FISH analysis.

### KD of Chd8 and other chromatin remodelers

In order to obtain *Chd8* KD, we used Dharmacon Smart pool-of-four siRNAs (#67772) or single siRNA (YY1, from Thermo Fisher, Cat #AM16708) at a 50 nM final concentration, in accordance with the manufacturers protocol. *Chd8* KD was initiated at day 1 of differentiation using lipofectamine transfection (RNAi-MAX Invitrogen).

In order to generate stable *Chd8* KD lines, we used lentiviral-mediated KD using pre-packaged lentiviral particles (SIGMA). In brief, Fa2L-S4 cell line was infected either with a scrambled shRNA control (SHC002) or two independent *Chd8*-targeting constructs (SIGMA shRNA clones—CloneIDs: NM_201637.2-847s1c1 (Chd8.1); NM_201637.2-3342s21c1 (Chd8.2)). Two days after transfection, puromycin selection was applied for 7–9 days (2 µg/ml). Colonies were picked and expanded using standard protocols. *Chd8* KD was assessed by conventional qRT-*PCR* analysis (see below).

### Generation of Chd8 KO cell lines

*Chd8* KO cells were generated by CRISPR-Cas9 mutagenesis. Briefly ~5 µg Cas9 expressing vector carrying Puromycin resistance and guides to the *Chd8* helicase and chromo domains were co-transfected in Fa2L-S4 cells using Lipofectamine 3000 (Invitrogen). Two days after transfection, puromycin selection was applied for 7–9 days (2 µg/ml). Colonies were picked and expanded using standard protocols. Clones surviving selection were screened by PCR for indels in either the chromo, the helicase or both domains. Amplicons were topocloned and analysed by DNA sequencing. We selected sub-clones C4.1/C4.3 for further analysis (see also Fig. 3 and the “Results” section).

### Complemented cell lines

We transfected *Chd8* KO clones by lipofection (Lipofectamine 3000, Invitrogen) in accordance with the manufacturer’s protocol. Briefly 10 µg of construct expressing the FL form of *Chd8* were transfected. Two days after transfection, Neomycin (G418) selection was applied for 7–9 days (400 µg/ml). Colonies were picked and expanded using standard protocols. Complementation was tested by means of WB analysis and immuno-fluorescence (IF) in the context of XCI initiation (see below and main text for details).

### RNA extraction and qRT-PCR analysis

RNA was extracted using the RNeasy mini kit according to the producer’s manual (Qiagen). RNA was DNAse treated for 30 min at 37 °C and DNAse was then inactivated using a cDNA kit (Turbo DNase, Ambion). Reverse-transcription was carried out using a kit from Thermo Scientific (First Strand cDNA Synthesis Kit, Cat. K1612) in accordance with the manufacturer’s instructions (1–2 µg of total RNA). qRT-PCR was performed using Kapa BioSystem/Biorad SYBR-green reagent using an Applied Biosystem real-time system, following standard protocols. A wide panel of primers pairs was used to test cell differentiation and XCI progression, in the initial phases of the project. Selected primers were then used for all experiments. A complete list of primers used in this paper is available in Supplementary Data 5. Primer sequences used in preliminary analysis (nor shown) are available on demand.

### Protein extraction and WB analysis

Whole lysate cell extracts were obtained after incubation on ice for 30′ in RIPA buffer with protease inhibitors, followed by centrifugation. WB analysis was done using standard SDS–PAGE protocols. CHD8 antibody(ab) was purchased from Novus biological (NB100-60417), HA ab (H6908), and Vin ab (V9264-200UL) both bought from Sigma, GAPDH ab bought from Millipore (CB1001), and RNA pol II ab obtained from Active Motif (#39097).

### Immunofluorescence

IF analysis and RNA-FISH were carried out as previously described^48^. H3K27me3 mono and polyclonal antibodies were purchased from Active Motif (#39155, #39536, respectively), *Chd8* antibodies were bought from Novus (NB100-60417).

### RNA-seq

RNA-seq analysis was carried out as previously described^8^. Reads were trimmed with *cutadapt* and aligned to the genome with STAR^70^. The *FeatureCounts* software counted the number of reads per gene overlapping any exon^71^. Genes are defined as in Ensembl’s genome release Mus Musculus GRCm38.88 (https://www.ensembl.org/Mus_musculus/Info/Index). DFG were identified using the Bioconductor package *DESeq2* in R version 3.6.2 requiring a FDR below 0.05 and an absolute log fold-change of at least 1 (https://www.bioconductor.org)^72^. Each analysis controlled for experimental batches and differences are reported as log2 fold-change with respect to the control group. Sequencing read counts were normalized using the regularized log transformation (rlog) in *DESeq2* before PCA. The top 20 DFG (by *P* value) are shown in the heatmaps. To ensure comparability across genes, read counts were normalized by variance stabilizing transform (vst) in *DESeq2* and mean normalized read counts per gene subtracted, before plotting the heatmap.

### ChIP-seq procedure and bioinformatic analysis

ChIP-seq analysis was carried out as previously published^73,74^. CHD8 antibodies were bought from Novus and Bethyl (NB100-60417 and A301-224A, respectively), H3K4me3 antibodies (Active Motif, Cat number 39159); INO80 ab was purchased from Abcam (Cat. Number: ab105451). Reads were trimmed with cutadapt (http://cutadapt.readthedocs.io/en/stable/index.html) and aligned to the genome with bowtie2 using the ‘very sensitive’ alignment option^75^. Picard tools were used to remove duplicate reads and samTools to filter out reads with mapping quality below two (https://broadinstitute.github.io/picard/). Subsequently, coverage plots were generated with deepTools using a bin size of 50 (or 100b) and RPKM (reads per kilobase per million) normalization^76^. The software MACS2 called peaks with 0.05 as FDR cut-off and consensus peak sets were derived for the duplicated experiments using Bioconductor’s package DiffBind (http://liulab.dfci.harvard.edu/MACS/; https://www.bioconductor.org).

### Comparison with other datasets

YY1 data was downloaded from the GEO repository (GSE92407). Bigwig files for YY1 (2i, 2 replicates) were converted to bedGraph. Peaks were called on the bedgraph files with MACS2. Consensus YY1 peaks were derived by intersecting the peaks called on the two replicates with bedtools. YY1 peaks were lifted from mm9 to mm10 using easyLift in R. YY1 and Chd8 peaks were overlapped using GenomicRanges in R. Peaks were annotated to genes (including distance to TSS) using the GREAT website (http://great.stanford.edu/public/html/). Promoter regions were defined as 0–2 kb upstream of the TSS, getting strand-specific information via biomaRt from the “m. musculus_gene_ensembl” dataset.

### ChIP- and ATAC-seq allelic analysis

ATAC-seq reads were processed and aligned as described elsewhere^77,78^. The software SNPsplit (https://www.bioinformatics.babraham.ac.uk/projects/SNPsplit/) was used for allele-specific analysis. In addition, to the alignment to the reference genome the reads were aligned to a genome in which all SNPs have been masked. Subsequently, aligned reads were divided into reads which either match CAST_EiJ or 129S1_SvImJ SNPs or if not were considered unspecific. Downstream analysis was done as before separately for each class of reads.

### Cut&Run experiments

This protocol was adapted from a CUT&RUN Protocol from EpiCypher (https://www.epicypher.com/content/documents/protocols/cutana-cut&run-protocol.pdf). Cells were harvested with accutase, counted and centrifuged at 600 × *g* for 3 min. Pellets were washed three times in 1.5 ml Wash buffer (20 mM HEPES pH 7.5; 150 mM NaCl; 0.5 mM Spermidine; 1× protease inhibitors EDTA-free) and resuspended in the right volume of Wash buffer in order to have 500,000 cells per 100 µl. 100 µl of the cell suspension were then transferred into an 8-stripe tube containing 10 µl of previously activated concanavalin A-coated magnetic beads (activating buffer: 20 mM HEPES pH 7.5, 10 mM KCl, 1 mM CaCl_2_, 1 mM MnCl_2_) (86057-3, BioMag^®^Plus Concanavalin A, Generon) and placed in a rotator for 10 min at room temperature. Cells were resuspended in 50 µl antibody buffer (5% (wt/vol) digitonin, 20 mM HEPES pH 7.5; 150 mM NaCl; 0.5 mM Spermidine; 1× protease inhibitors EDTA-free, 2 mM EDTA) and 0.5 µl antibody was added per sample and incubated at 4 °C overnight in a rotator. The following antibodies were used: YY1 (H-10): Santa Cruz sc-7341; YY1 Diagenode AB_2793763; WDR5: Diagenode C15310101. Cells were washed 3× in 250 µl Dig-wash buffer (5% (wt/vol) digitonin, 20 mM HEPES pH 7.5; 150 mM NaCl; 0.5 mM Spermidine; 1× protease inhibitors EDTA-free) to remove unbound antibody. Cells were resuspended in 50 µl cold Dig-wash buffer and pAG-MNase 1:200 was added and incubated at room temperature for 10 min At this stage, supernatant from samples incubated with IgG antibody was collected and stored to be used as input. Cells were washed 3× in 250 µl cold Dig-wash buffer to remove unbound pAG-MNase. Tubes were placed on ice and quickly mixed with 100 mM CaCl_2_ to a final concentration of 2 mM diluted in 50 µl Dig-wash buffer per sample. Cells were incubated for 2 h at 4 °C and the reaction was quenched by the addition of 33 µl 2× STOP buffer (340 mM NaCl; 20 mM 0.5 EDTA; 4 mM EGTA; 0.05% digitonin; 100 µg/mL RNAse A; 50 µg/mL glycogen). Cleaved fragments were liberated into the supernatant by incubating the cells at 37 °C for 10 min, and the supernatant was stored afterwards. DNA fragments were purified from the supernatant using the ChIP DNA Clean and Concentrator Kit (D5205, Zymo Research) and used for the construction of sequencing libraries.

### MEME analysis and string

MEME analysis was done by extracting 100 and 500 bp windows underlying *Chd8* peaks in undifferentiated and differentiating conditions. These sequences have been analysed using MEME motif discovery via the weblink (http://meme-suite.org/). In order to discover *Chd8*-interacting proteins, we used the online version of the STRING database (https://string-db.org/).

### Chd8 IP-mass spectrometry

CHD8 and HA IP were done using *Chd8* antibodies (abs) bought from Novus biological (NB100-60417) and Bethyl (A301-224A), HA ab was acquired from Sigma (H6908). For each sample 500 µl of extracted lysate was brought to 1 ml with RIPA buffer and precleared with 50 µl Protein G Dynabeads (Thermo) for 30 min on a rotating wheel at 4 °C. Beads were removed and 15 µl of antibodies was added to the cleared lysate and incubated at 4 °C for an hour on the rotating wheel, after which 50 µl of washed protein G beads were added and incubated for another hour. Beads were washed three times with 250 µl RIPA buffer and eluted in 20 µl Laemmle buffer by boiling for 5 min Samples were run on a 10% SDS–PAGE gel and analysed by mass spectrometry. Protein and RNA sequences were downloaded from UCSC genome browser and Uniprot, respectively (https://genome.ucsc.edu/; https://www.uniprot.org/).

### LC–MS/MS

Bands were cut from the gel and subjected to in-gel digestion with trypsin^79^. Peptides were extracted from the gel pieces by sonication for about 15 min, followed by centrifugation and the collection of the supernatant. A 50:50 solution of water:acetonitrile, 1% formic acid (2 × the volume of the gel pieces) was added for a second extraction. The samples were re-sonicated for 15 min, centrifuged and the supernatant pooled with the first extract. The pooled supernatants were processed using speed vacuum centrifugation. The samples were dissolved in 10 µL of reconstitution buffer (96:4 water:acetonitrile, 1% formic acid and analyzed by LC–MS/MS.

Peptides were separated using the nanoAcquity UPLC system (Waters) fitted with a trapping devise (nanoAcquity Symmetry C18, 5 µm, 180 µm × 20 mm) and an analytical column (nanoAcquity BEH C18, 1.7 µm, 75 µm × 200 mm). The outlet of the analytical column was coupled to an LTQ Orbitrap Velos (Thermo Fisher Scientific) using the Proxeon nanospray source. Solvent A was water, 0.1% formic acid and solvent B was acetonitrile, 0.1% formic acid. Samples were loaded with a constant flow of solvent A at 5 µL/min onto the trapping column, as routinely done. Trapping time was 6 min Peptides were eluted via the analytical column a constant flow of 0.3 µL/min. During the elution step, the percentage of solvent B was increased in a linear fashion. The peptides were introduced into the mass spectrometer instrument (Orbitrap Velos, Thermo) via a Pico-Tip Emitter 360 μm OD × 20 μm ID; 10 μm tip (New Objective) and a spray voltage of 2.2 kV was applied. The capillary temperature was set at 300 °C. Full scan MS spectra with mass range 300–1700 m/z were acquired in profile mode in the FT with a resolution of 30,000. The filling time was set at maximum of 500 ms with limitation of 1.0 × 10^6^ ions. The most intense ions (up to 15) from the full scan MS were selected for sequencing in the LTQ. Normalized collision energy of 40% was used, and the fragmentation was performed after accumulation of 3.0 × 10^4^ ions or after filling time of 100 ms for each precursor ion (whichever occurred first). MS/MS data was acquired in centroid mode. Only multiply charged (2+, 3+, 4+) precursor ions were selected for MS/MS analysis. The dynamic exclusion list was restricted to 500 entries with maximum retention period of 30 s and relative mass window of 10 ppm. In order to improve the mass accuracy, a lock mass correction using the ion (*m*/*z* 445.12003) was applied.

### MS data analysis

The raw output files of IsobarQuant (protein.txt–files) were processed using the R programming language (ISBN 3-900051-07-0). Only proteins that were quantified with at least two unique peptides were considered for the analysis. Raw TMT reporter ion signals (signal_sum columns) were first cleaned for batch effects using the ‘removeBatchEffect’ function of the limma package^80^ and further normalized using vsn (variance stabilization normalization)^81^. Proteins were tested for differential expression using the limma package. A protein was annotated as a hit with a FDR smaller 5% and a fold-change of at least 100% and as a candidate with a fdr below 10% and a fold-change of at least 50%.

### Statistical reproducibility

Conventional two-tailed *t*-test and Poisson regression fittings were used in R environment (https://www.r-project.org/) or JMP software (jmp.com) or excel (Microsoft). All experimental data points are clearly presented in the figure legends or in the figure the statistical test used and the error bar type is clearly indicated. No statistical test was performed on log-scaled values.

### Reporting summary

Further information on research design is available in the Nature Research Reporting Summary linked to this article.

## Supplementary information

Peer Review File

Supplementary Information

Description of Additional Supplementary Files

Supplementary Data 1

Supplementary Data 2

Supplementary Data 3

Supplementary Data 4

Supplementary Data 5

Supplementary Data 6

Reporting Summary

## Data Availability

Next-generation sequencing data has been deposited in GEO, Access numbers: GSE166858 and GSE166859. All source data underlying the graphs and charts presented in the main figures are available in Supplementary Data 6. Mass-spectrometry data has been deposited in the PRIDE database, accession number: PXD024155. All data is available upon request.

## References

[1] Robert Finestra T, Gribnau J (2017). X chromosome inactivation: silencing, topology and reactivation. Curr. Opin. Cell Biol..

[2] Gribnau J, Grootegoed JA (2012). Origin and evolution of X chromosome inactivation. Curr. Opin. Cell Biol..

[3] Cerase A, Pintacuda G, Tattermusch A, Avner P (2015). Xist localization and function: new insights from multiple levels. Genome Biol..

[4] Pinter SF (2016). A Tale of Two Cities: how Xist and its partners localize to and silence the bicompartmental X. Semin. Cell Dev. Biol..

[5] Schulz EG, Heard E (2013). Role and control of X chromosome dosage in mammalian development. Curr. Opin. Genet. Dev..

[6] Almeida M (2017). PCGF3/5-PRC1 initiates Polycomb recruitment in X chromosome inactivation. Science.

[7] Pintacuda G (2017). hnRNPK recruits PCGF3/5-PRC1 to the Xist RNA B-Repeat to establish polycomb-mediated chromosomal silencing. Mol. Cell.

[8] Pintacuda G, Young AN, Cerase A (2017). Function by structure: spotlights on Xist long non-coding RNA. Front. Mol. Biosci..

[9] Moindrot B (2015). A pooled shRNA screen identifies Rbm15, Spen, and Wtap as factors required for Xist RNA-mediated silencing. Cell Rep..

[10] Cirillo D (2016). Quantitative predictions of protein interactions with long noncoding RNAs. Nat. Methods.

[11] Pinter SF (2012). Spreading of X chromosome inactivation via a hierarchy of defined Polycomb stations. Genome Res..

[12] Cerase, A. et al. Phase separation drives X-chromosome inactivation: a hypothesis. *Nat. Struct. Mol. Biol.*10.1038/s41594-019-0223-0 (2019). 10.1038/s41594-019-0223-0 31061525

[13] Cerase A, Tartaglia GG (2020). Long non-coding RNA-polycomb intimate rendezvous. Open Biol..

[14] Chaumeil J, Le Baccon P, Wutz A, Heard E (2006). A novel role for Xist RNA in the formation of a repressive nuclear compartment into which genes are recruited when silenced. Genes Dev..

[15] Heard E (2004). Recent advances in X-chromosome inactivation. Curr. Opin. Cell Biol..

[16] Pintacuda G, Cerase A (2015). X inactivation lessons from differentiating mouse embryonic stem cells. Stem Cell Rev..

[17] Navarro P (2008). Molecular coupling of Xist regulation and pluripotency. Science.

[18] Navarro P (2009). A role for non-coding Tsix transcription in partitioning chromatin domains within the mouse X-inactivation centre. Epigenet. Chromatin.

[19] Navarro P (2010). Molecular coupling of Tsix regulation and pluripotency. Nature.

[20] Navarro P, Page DR, Avner P, Rougeulle C (2006). Tsix-mediated epigenetic switch of a CTCF-flanked region of the Xist promoter determines the Xist transcription program. Genes Dev..

[21] Navarro P, Pichard S, Ciaudo C, Avner P, Rougeulle C (2005). Tsix transcription across the Xist gene alters chromatin conformation without affecting Xist transcription: implications for X-chromosome inactivation. Genes Dev..

[22] Tian D, Sun S, Lee JT (2010). The long noncoding RNA, Jpx, is a molecular switch for X chromosome inactivation. Cell.

[23] Sun BK, Deaton AM, Lee JT (2006). A transient heterochromatic state in Xist preempts X inactivation choice without RNA stabilization. Mol. Cell.

[24] Barakat TS (2014). The trans-activator RNF12 and cis-acting elements effectuate X chromosome inactivation independent of X-pairing. Mol. Cell.

[25] Gontan C (2012). RNF12 initiates X-chromosome inactivation by targeting REX1 for degradation. Nature.

[26] Chureau C (2011). Ftx is a non-coding RNA which affects Xist expression and chromatin structure within the X-inactivation center region. Hum. Mol. Genet..

[27] Furlan G (2018). The Ftx noncoding locus controls X chromosome inactivation independently of its RNA products. Mol. Cell.

[28] Augui S, Nora EP, Heard E (2011). Regulation of X-chromosome inactivation by the X-inactivation centre. Nat. Rev. Genet..

[29] van Bemmel JG, Mira-Bontenbal H, Gribnau J (2016). Cis- and trans-regulation in X inactivation. Chromosoma.

[30] Azuara V (2006). Chromatin signatures of pluripotent cell lines. Nat. Cell Biol..

[31] Clapier CR, Iwasa J, Cairns BR, Peterson CL (2017). Mechanisms of action and regulation of ATP-dependent chromatin-remodelling complexes. Nat. Rev. Mol. Cell Biol..

[32] Narlikar GJ, Sundaramoorthy R, Owen-Hughes T (2013). Mechanisms and functions of ATP-dependent chromatin-remodeling enzymes. Cell.

[33] Ho L, Crabtree GR (2010). Chromatin remodelling during development. Nature.

[34] Gao X (2008). ES cell pluripotency and germ-layer formation require the SWI/SNF chromatin remodeling component BAF250a. Proc. Natl Acad. Sci. USA.

[35] Marfella CG (2006). Mutation of the SNF2 family member Chd2 affects mouse development and survival. J. Cell. Physiol..

[36] de Dieuleveult M (2016). Genome-wide nucleosome specificity and function of chromatin remodellers in ES cells. Nature.

[37] Casper, J. et al. The UCSC Genome Browser database: 2018 update. *Nucleic Acids Res.*10.1093/nar/gkx1020 (2017). 10.1093/nar/gkx1020 PMC5753355 29106570

[38] Zerbino, D. R. et al. Ensembl 2018. *Nucleic Acids Res.*10.1093/nar/gkx1098 (2017).

[39] Manning BJ, Yusufzai T (2017). The ATP-dependent chromatin remodeling enzymes CHD6, CHD7, and CHD8 exhibit distinct nucleosome binding and remodeling activities. J. Biol. Chem..

[40] Nishiyama M (2004). Early embryonic death in mice lacking the beta-catenin-binding protein Duplin. Mol. Cell. Biol..

[41] Wilkinson B (2015). The autism-associated gene chromodomain helicase DNA-binding protein 8 (CHD8) regulates noncoding RNAs and autism-related genes. Transl. Psychiatry.

[42] Breuss MW, Gleeson JG (2016). When size matters: CHD8 in autism. Nat. Neurosci..

[43] Gompers AL (2017). Germline Chd8 haploinsufficiency alters brain development in mouse. Nat. Neurosci..

[44] Katayama Y (2016). CHD8 haploinsufficiency results in autistic-like phenotypes in mice. Nature.

[45] Xu Q (2018). Autism-associated CHD8 deficiency impairs axon development and migration of cortical neurons. Mol. Autism.

[46] Luikenhuis S, Wutz A, Jaenisch R (2001). Antisense transcription through the Xist locus mediates Tsix function in embryonic stem cells. Mol. Cell. Biol..

[47] Doran AG (2016). Deep genome sequencing and variation analysis of 13 inbred mouse strains defines candidate phenotypic alleles, private variation and homozygous truncating mutations. Genome Biol..

[48] Cerase A (2014). Spatial separation of Xist RNA and polycomb proteins revealed by superresolution microscopy. Proc. Natl Acad. Sci. USA.

[49] Ceballos-Chavez M (2015). The chromatin Remodeler CHD8 is required for activation of progesterone receptor-dependent enhancers. PLoS Genet..

[50] Cohen DE (2007). The DXPas34 repeat regulates random and imprinted X inactivation. Dev. Cell.

[51] Cotney J (2015). The autism-associated chromatin modifier CHD8 regulates other autism risk genes during human neurodevelopment. Nat. Commun..

[52] Sugathan A (2014). CHD8 regulates neurodevelopmental pathways associated with autism spectrum disorder in neural progenitors. Proc. Natl Acad. Sci. USA.

[53] Memili E, Hong YK, Kim DH, Ontiveros SD, Strauss WM (2001). Murine Xist RNA isoforms are different at their 3′ ends: a role for differential polyadenylation. Gene.

[54] Ma M, Strauss WM (2005). Analysis of the Xist RNA isoforms suggests two distinctly different forms of regulation. Mamm. Genome.

[55] Nora EP (2017). Targeted degradation of CTCF decouples local insulation of chromosome domains from genomic compartmentalization. Cell.

[56] Makhlouf M (2014). A prominent and conserved role for YY1 in Xist transcriptional activation. Nat. Commun..

[57] Wang L (2018). Resetting the epigenetic balance of Polycomb and COMPASS function at enhancers for cancer therapy. Nat. Med..

[58] Zurkirchen L (2019). Yin Yang 1 sustains biosynthetic demands during brain development in a stage-specific manner. Nat. Commun..

[59] Beagan JA (2017). YY1 and CTCF orchestrate a 3D chromatin looping switch during early neural lineage commitment. Genome Res..

[60] Loos F (2016). Xist and Tsix transcription dynamics is regulated by the X-to-autosome ratio and semistable transcriptional states. Mol. Cell. Biol..

[61] Pontier DB, Gribnau J (2011). Xist regulation and function explored. Hum. Genet..

[62] Sun S (2013). Jpx RNA activates Xist by evicting CTCF. Cell.

[63] Chen CY (2016). YY1 binding association with sex-biased transcription revealed through X-linked transcript levels and allelic binding analyses. Sci. Rep..

[64] Atlasi Y (2019). Epigenetic modulation of a hardwired 3D chromatin landscape in two naive states of pluripotency. Nat. Cell Biol..

[65] Subtil-Rodriguez A (2014). The chromatin remodeller CHD8 is required for E2F-dependent transcription activation of S-phase genes. Nucleic Acids Res..

[66] Nishiyama M, Skoultchi AI, Nakayama KI (2012). Histone H1 recruitment by CHD8 is essential for suppression of the Wnt-beta-catenin signaling pathway. Mol. Cell. Biol..

[67] Durak O (2016). Chd8 mediates cortical neurogenesis via transcriptional regulation of cell cycle and Wnt signaling. Nat. Neurosci..

[68] Marks H (2012). The transcriptional and epigenomic foundations of ground state pluripotency. Cell.

[69] Attia M, Rachez C, Avner P, Rogner UC (2013). Nucleosome assembly proteins and their interacting proteins in neuronal differentiation. Arch. Biochem. Biophys..

[70] Dobin A (2013). STAR: ultrafast universal RNA-seq aligner. Bioinformatics.

[71] Liao Y, Smyth GK, Shi W (2014). featureCounts: an efficient general purpose program for assigning sequence reads to genomic features. Bioinformatics.

[72] Love MI, Huber W, Anders S (2014). Moderated estimation of fold change and dispersion for RNA-seq data with DESeq2. Genome Biol..

[73] Farcas AM (2012). KDM2B links the Polycomb Repressive Complex 1 (PRC1) to recognition of CpG islands. eLife.

[74] De Bonis ML (2006). Maintenance of X- and Y-inactivation of the pseudoautosomal (PAR2) gene SPRY3 is independent from DNA methylation and associated to multiple layers of epigenetic modifications. Hum. Mol. Genet..

[75] Langmead B, Salzberg SL (2012). Fast gapped-read alignment with Bowtie 2. Nat. Methods.

[76] Li H (2009). The Sequence Alignment/Map format and SAMtools. Bioinformatics.

[77] Buenrostro JD, Giresi PG, Zaba LC, Chang HY, Greenleaf WJ (2013). Transposition of native chromatin for fast and sensitive epigenomic profiling of open chromatin, DNA-binding proteins and nucleosome position. Nat. Methods.

[78] Corces MR (2017). An improved ATAC-seq protocol reduces background and enables interrogation of frozen tissues. Nat. Methods.

[79] Savitski MM (2014). Tracking cancer drugs in living cells by thermal profiling of the proteome. Science.

[80] Ritchie ME (2015). limma powers differential expression analyses for RNA-sequencing and microarray studies. Nucleic Acids Res..

[81] Huber W, von Heydebreck A, Sultmann H, Poustka A, Vingron M (2002). Variance stabilization applied to microarray data calibration and to the quantification of differential expression. Bioinformatics.

