# Supplementary material for: Chd8 regulates X chromosome inactivation in mouse through fine-tuning control of Xist expression
Source: Commun Biol. 2021 Apr 15;4:485. doi: 10.1038/s42003-021-01945-1 (PMC8050208; doi:10.1038/s42003-021-01945-1)
Supplement: Supplementary file 2 — Supplementary Information [file 42003_2021_1945_MOESM2_ESM.pdf]

Fig. S1: *Fa2l-S4 cell line is a suitable system to study the initiation of XCI*

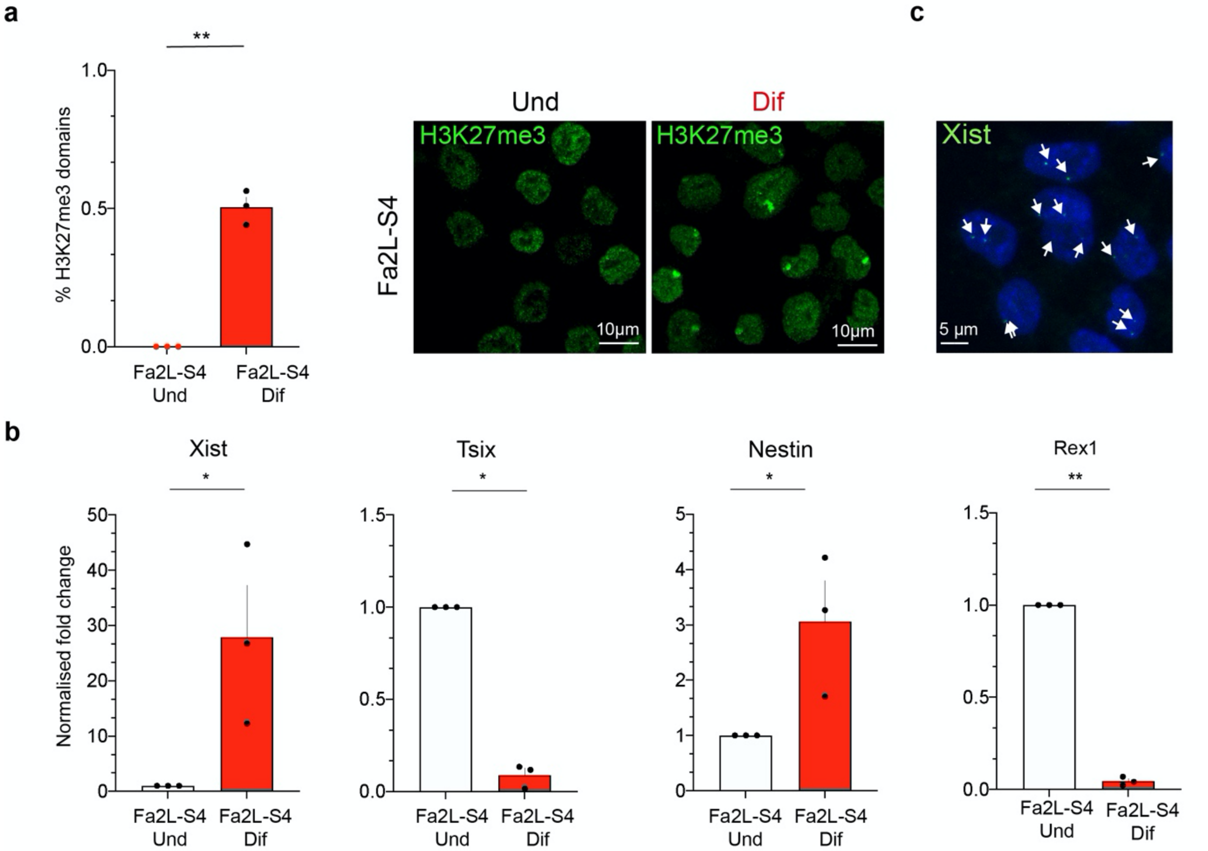

a) Left: quantitation of H3K27me3 domains by IF in Fa2L-S4 cell lines at three-day differentiation, right: representative images. Data from three experiments is shown, n>1000. Single points represent independent biological samples. p-value=0.00494 (Und vs Dif). **b)** XCI and cell differentiation are properly initiated in Fa2L-S4 cell line (qPCR data). *Nestin* is used as an NPC differentiation marker and *Rex1* as marker of pluripotency and exit from the pluripotent state. Undifferentiated and differentiated conditions are shown (Und/Dif). Data from three experiments is shown. Single points represent independent biological samples. Statistical significance was tested by means of two-tailed unpaired t-test (p*≤0.05). p-values, Rex1=0.000205; Tsix=0.00167. Gapdh was used as internal normalization control. Data is normalised for the undifferentiated condition (Und). **c)** DNA-FISH in Fa2L-S4 shows two X chromosomes. Xist cDNA has been used a probe (green). White arrows indicated the Xist gene.

Fig. S2*: A representative example of Chd8 and H3K4me co-localization at promoter regions by ChIP-seq analysis*

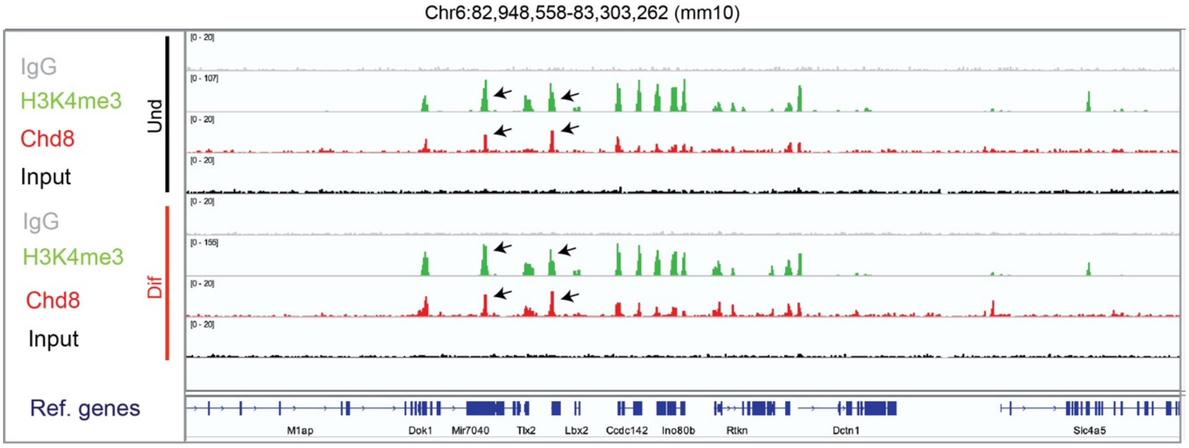

Chr. 6: 83,085,264-83,439,968 interval is shown (mm10). Red: Chd8; green: H3K4me3; grey: IgG; black: input. Undifferentiated and differentiated conditions are shown (Und/Dif). One representative set of ChIP-seq profiles is shown. Arrows show random examples of Chd8 and H3K4me3 co-localization. Ref. genes are shown in blue. Samples and antibodies used are shown.

Fig. S3: *Allelic analysis of the Xist promoter for H3K4me3 and Chd8*

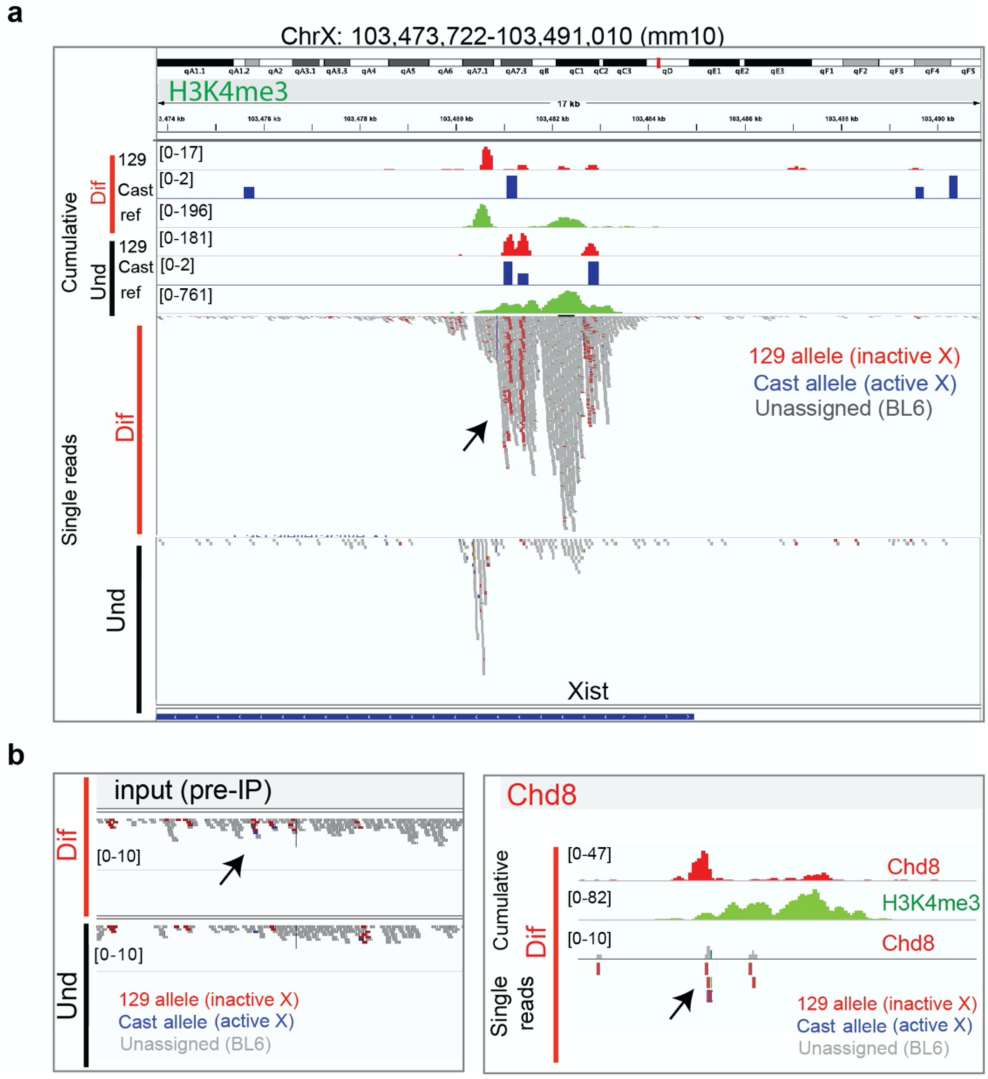

a) Chr. X: 103,473,722-103,491,010 interval is shown (mm10) Red line: three day differentiated cells (Dif); black line undifferentiated cells (Und). Control line Fa2L-S4 is shown. Samples names and genomes are shown. Allelic calls for the *129* genome (129, red) or the *m.castaneus* genome (Cast, blue); Ref genome, C57Black/6 (BL6, grey), are shown. **b)** Left, zoom-in on the Xist promoter for the input sample shows no allelic differences in the starting Materials (pre-IP conditions). One representative profile is shown. Right, allelic call for Chd8 in differentiating conditions at the Xist promoter. Blue: *castaneus* allele (Cast), Red: *129* allele, Grey: *BL6* genome (unassigned, Ref genome). One representative profile is shown.

Fig. S4: ***Xist and Chd8 expression during differentiation***

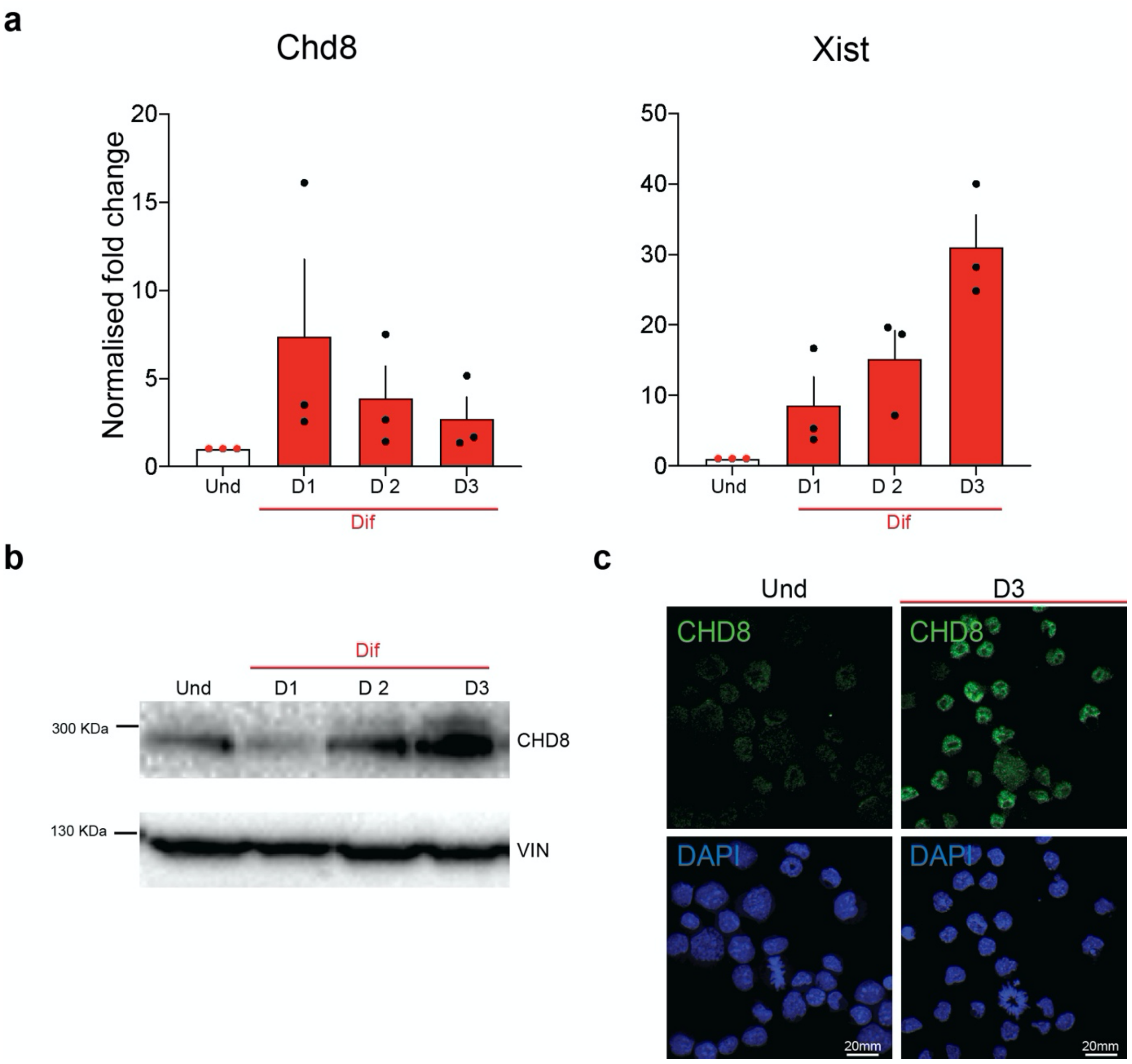

a) *Xist* and *Chd8* expression at RNA level is shown (qRT-PCR data). Error bar represents standard deviation of the mean (SEM). Three biological replicas are shown. Data is normalised for the undifferentiated condition (Und). Single points represent independent biological samples. Gapdh was used as internal normalization control. **b)** WB analysis of CHD8 (top); vinculin (VIN) was used as a loading control (bottom). Representative images are shown. **c)** IF for CHD8 at day three of differentiation in Fa2L-S4 cells is shown. D3 indicated 3 days of differentiation.

Fig. S5: ***The onset of XCI is partially compromised in mild Chd8 KD experiments***

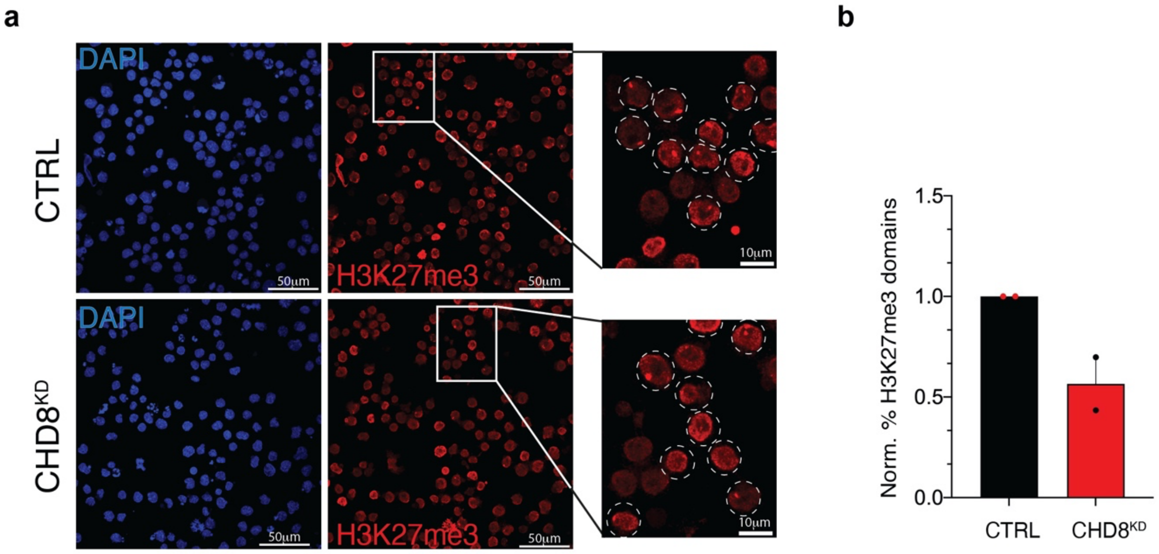

a) Representative images of H3K27me3 in CTRL vs. Chd8^{KD} cells. **b)** CTRL-normalised % of H3K27me3 domains in CTRL vs. Chd8^{KD} cells is shown, n=904. Error bars represent standard deviation (SD). Two experiments are shown. Single points represent independent biological samples.

Fig. S6: ***RNA-seq data analysis shows little expression variation in Chd8 KD at three-day differentiation (siRNA)***

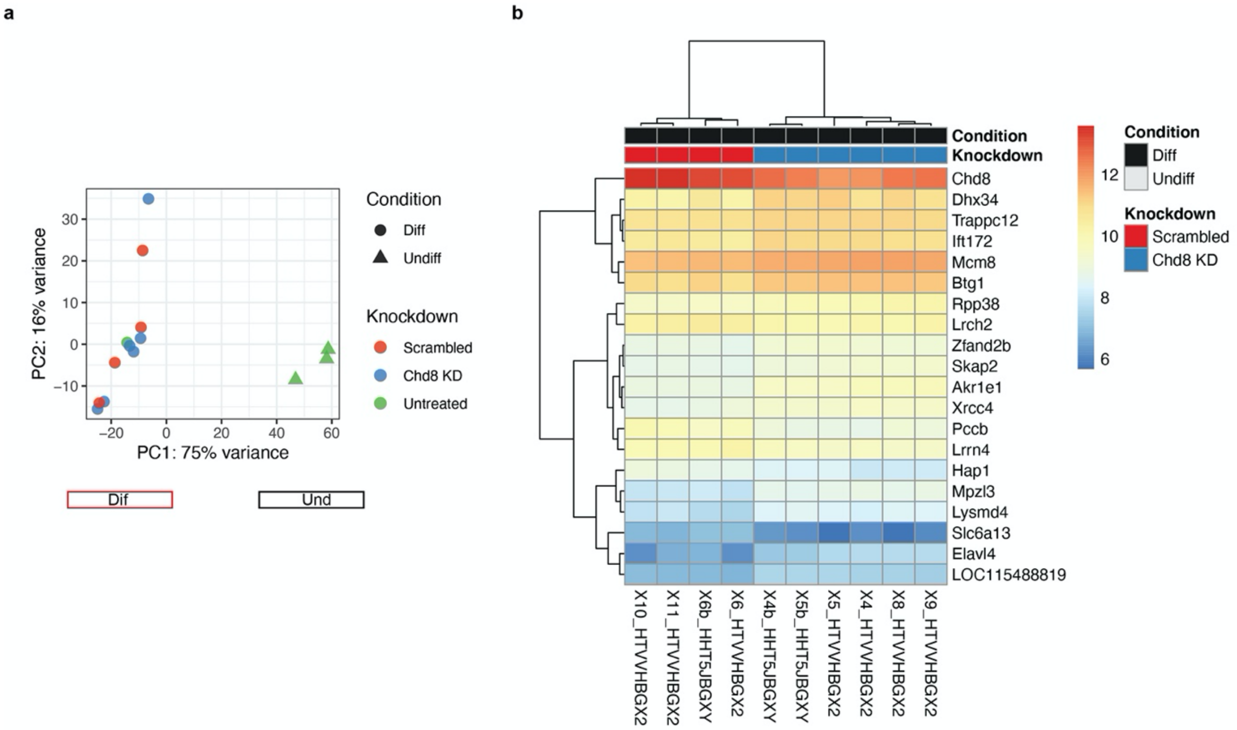

a) PCA plot of differentiated cells vs. undifferentiated cells. In blue the Chd8 KD, in red the scrambled control (CTRL) and in green untreated cells. Triangles and circles indicate undifferentiated and untreated cells (Und - Dif), respectively. **b)** Clustered heatmap graph, showing very modest differences in gene expression under Chd8 KD.

Fig. S7: *Analysis of shRNA-mediated Chd8 KD in the context of X chromosome inactivation*

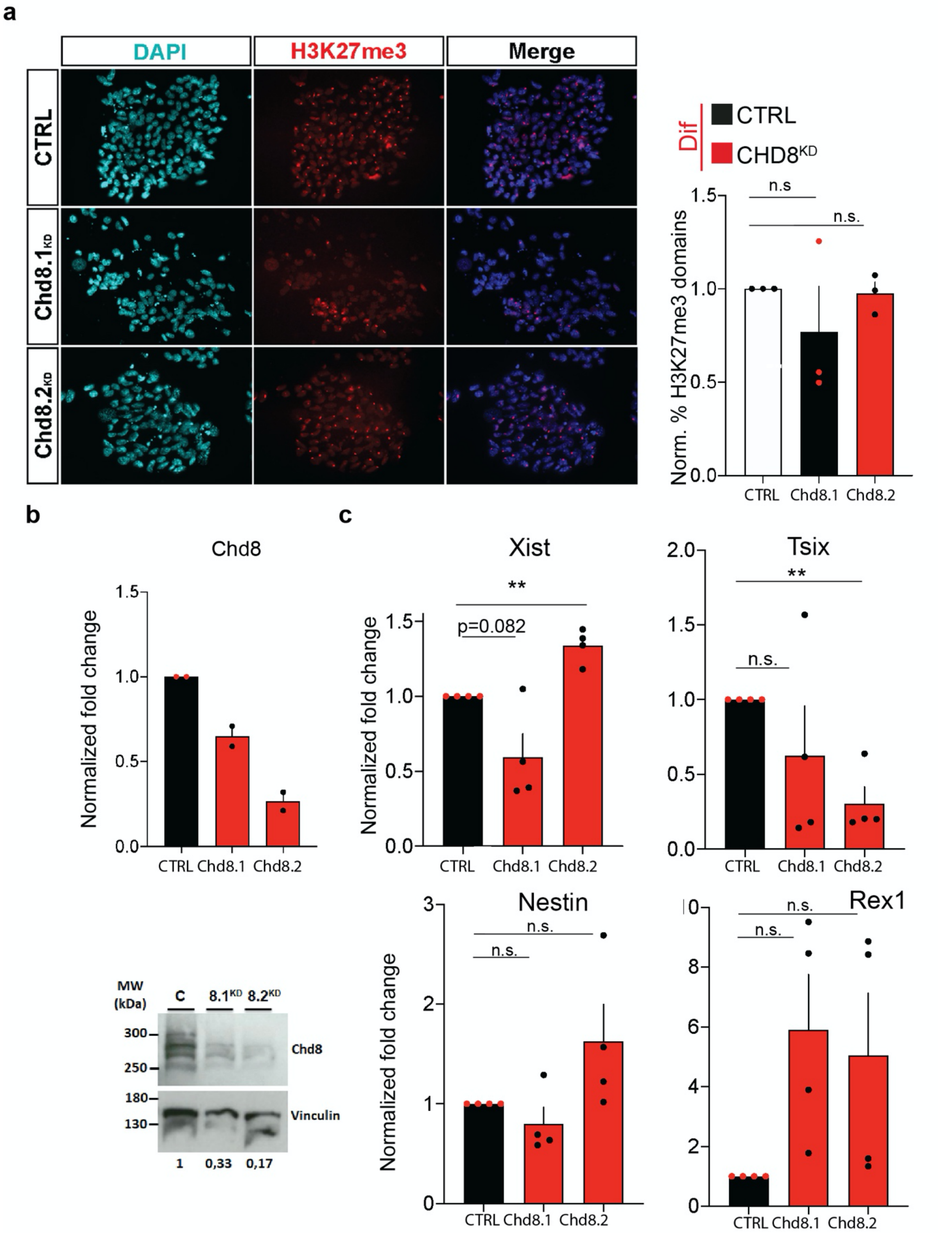

a) Representative images of H3K27me3 IFs in CTRL vs. Chd8 KD cells at three days of differentiation (mild and severe Chd8 KDs are show: Chd8.1 and Chd8.2, respectively). Normalised percentage of H3K27me3 domains in CTRL vs. Chd8 KD cells is shown (right), n>1000. Single points represent independent biological samples. Error bars represent standard deviation of the mean (SEM). Data comes from three independent experiments. **b)** Top, qRT-PCR of Chd8 KD cell lines vs. parental (Fa2l-S4). Gapdh was used as internal normalization control. Bottom, Western Blot analysis for CHD8 and Vinculin (VCL) (below, normalised quantifications in bold). A representative image is shown. **c)** CTRL-normalised qRT-PCR of XCI and differentiation markers as shown before. Data comes from two independent experiments. Error bars represent standard deviation of the mean (SEM). Statistical significance was tested by means of two-tailed unpaired t-test (p*≤0.05). Gapdh was used as internal normalization control. Single points represent independent biological samples. Statistical significance was tested by means of two-tailed unpaired t-test (p*≤0.05). Tsix Chd8.2 p-value= 0.00828, Xist Chd8.2 p=0.0096.

Fig. S8: ***RNA-seq data analysis shows gene expression variation differences in mild vs. severe Chd8 KD***

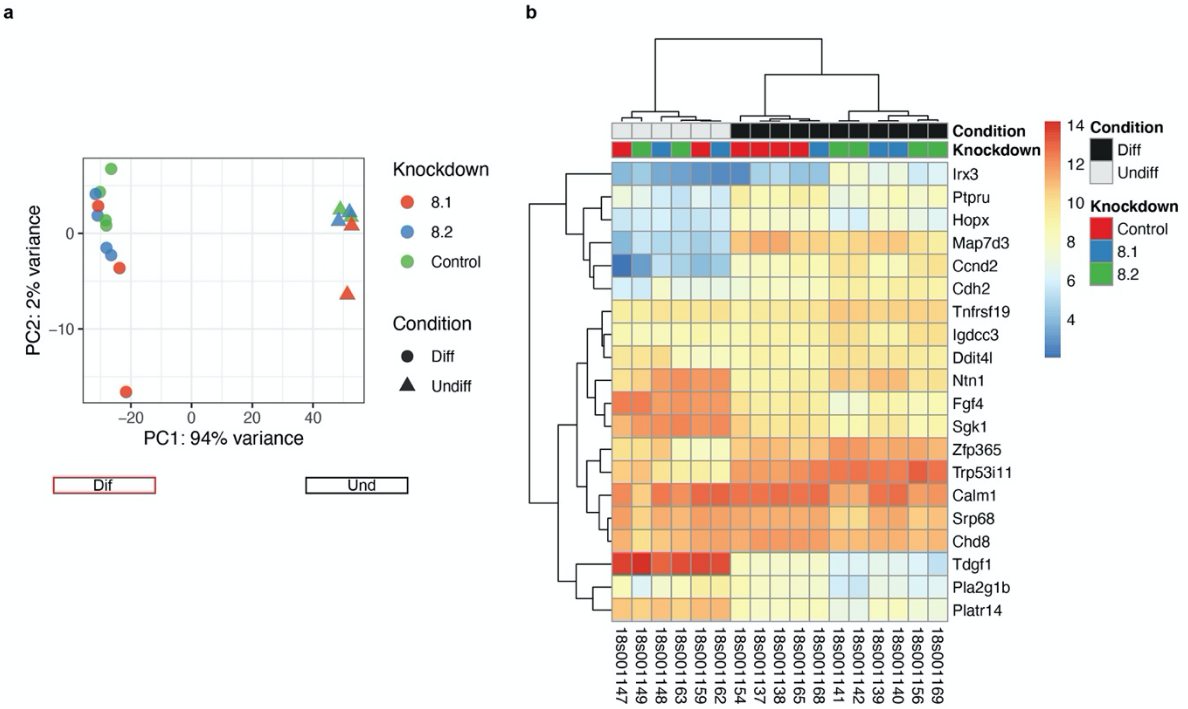

a) PCA plot of differentiated (red box) cells vs. undifferentiated (black box) cells. The Chd8 KD.2 is shown in blue, the Chd8 KD.1 is shown in red and the scrambled control (CTRL), in green. Triangles and circles indicate undifferentiated and untreated cells (Und - Dif), respectively **b)** Clustered heatmap graph of normalized RNA-seq reads, showing Chd8.2 KD vs Control. The top 20 significantly differentially expressed genes were included in the heatmap.

Fig. S9: *Bioinformatic and Mass-spectrometry analysis of Chd8 motifs and Chd8 interacting partners*

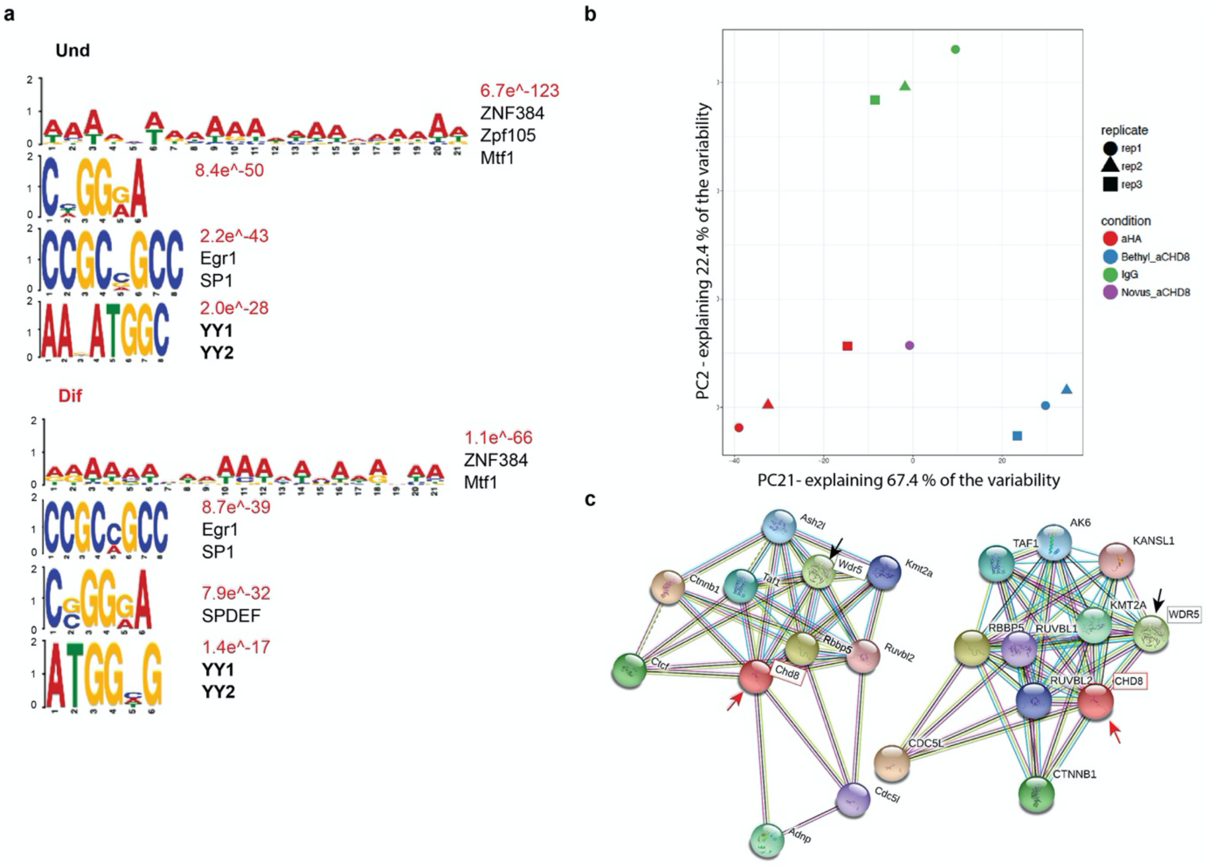

a) MEME analysis of Chd8 peaks in undifferentiated and differentiated conditions (top, bottom – respectively). **b)** PCA plot of Mass Spectrometry data. Replicas are shown as circles, triangles and squares. Anti-HA (aHA, red), Anti-Chd8 (Bethyl_aCHD8, Blue), Anti-IgG (IgG, green), Anti-Chd8 (Novus_aCHD8, purple). **C)** STRING analysis of Chd8 interactions in mouse (left) and human (right). Wdr5/WDR5 is also indicated (black arrow/box), Chd8 is highlighted (red box/red arrow).

Fig. S10: *Generation and characterization of a Chd8 knock-in cell line*

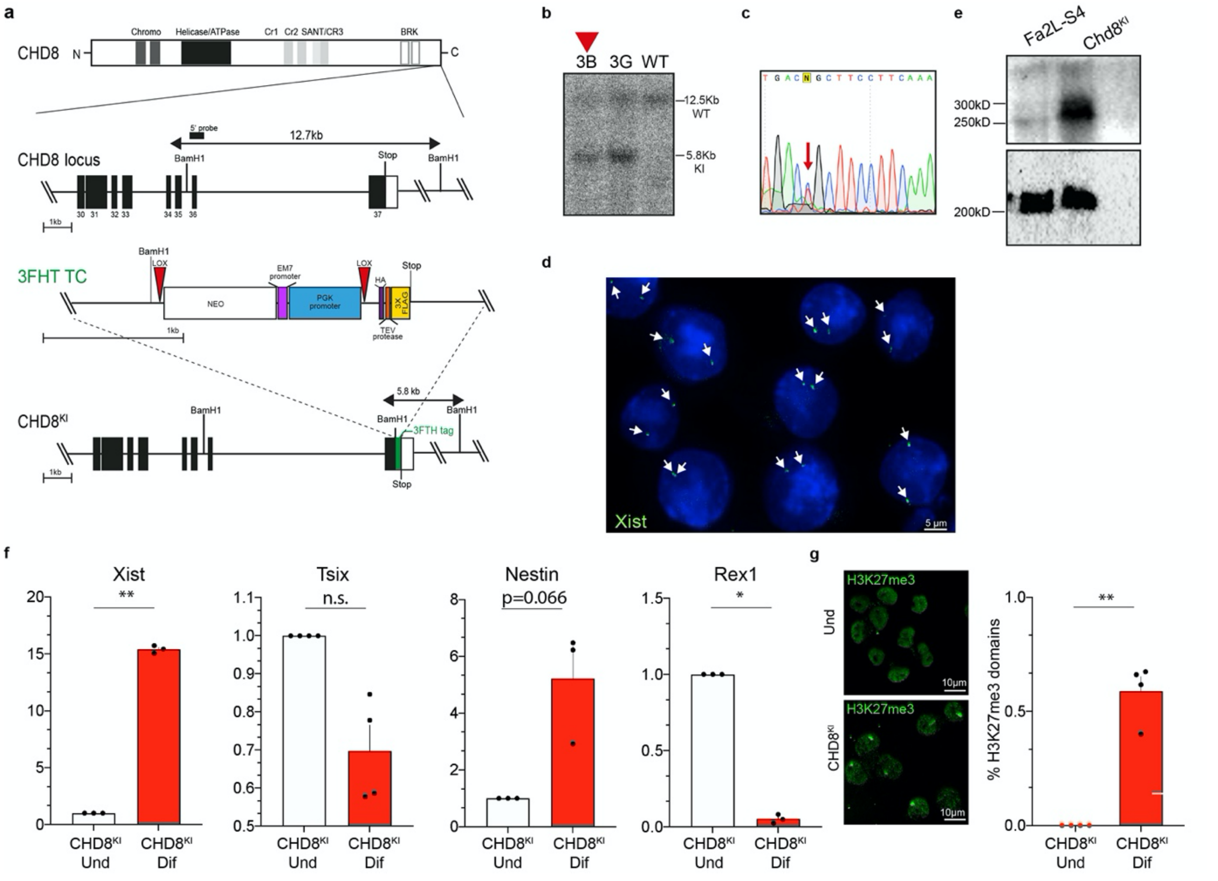

a) The domain structure of the CDH8 protein is shown. Below the organization of the 3' portion of the Chd8 locus and the targeting vector for introducing the 3FHT (3Flag-HA-TAG) into the Chd8 locus is depicted. Bottom) schematic map of the targeted *Chd8* gene, after targeting vector insertion, is shown. Triangles represent loxP sites. Rectangles indicate the position of Neomycin (Neo cassette, white), EM7 promoter (purple), Pgk promoter (blue), HA tag (violet), TEV protease (orange) and the three flag tags (yellow). The BamH1 restriction sites are indicated as well as the respective Southern fragments detected by the 5′ probe. **b)** Southern blot of BamH1-digested DNA from wild-type and CHD8-3FTH/WT cells (clones 3B and 3G) are shown (5′ probe indicated in a). **c)** A chromatogram shows a randomly-selected *129/castaneus* SNP (rs33716600) **d)** *Xist* DNA-FISH shows the two X chromosomes present in most cell **e)** WB showing that the tagged allele is expressed in the Chd8^{KI} line. Top HA antibody (ab) and bottom RNA-polII ab. **f)** XCI and cell differentiation are properly initiated in the Chd8 KI cell line (qPCR-data is shown). Data is normalised for the undifferentiated condition (Und)). *Nestin* is used as an NPC differentiation marker and *Rex1* as marker

of pluripotency and exit from the pluripotent state. Undifferentiated and differentiated conditions are shown (Und/Dif). Data from three experiments is shown. Four experiments are shown for Tsix. Single points represent independent biological samples. Statistical significance was tested by means of two-tailed unpaired t-test ($p^* \leq 0.05$). p-values, Rex1=0.000304; Xist=0.000186. Gapdh was used as internal normalization control. **g)** Left, representative images of H3K27me3 in CTRL vs. Chd8^{KI} cells. Right, % of H3K27me3 domains in CTRL vs. Chd8^{KI} cells is shown, n=476. Error bars represent standard deviation of the mean (SEM). Three experiments are shown. Statistical significance was tested by means of two-tailed unpaired t-test ($p^* \leq 0.05$). p-value= 0.00270 (Und vs. Dif). Single points represent independent biological samples.

Fig. S11) *ChIP-seq and Cut&Run qPCR analysis of candidate Xist regulators*

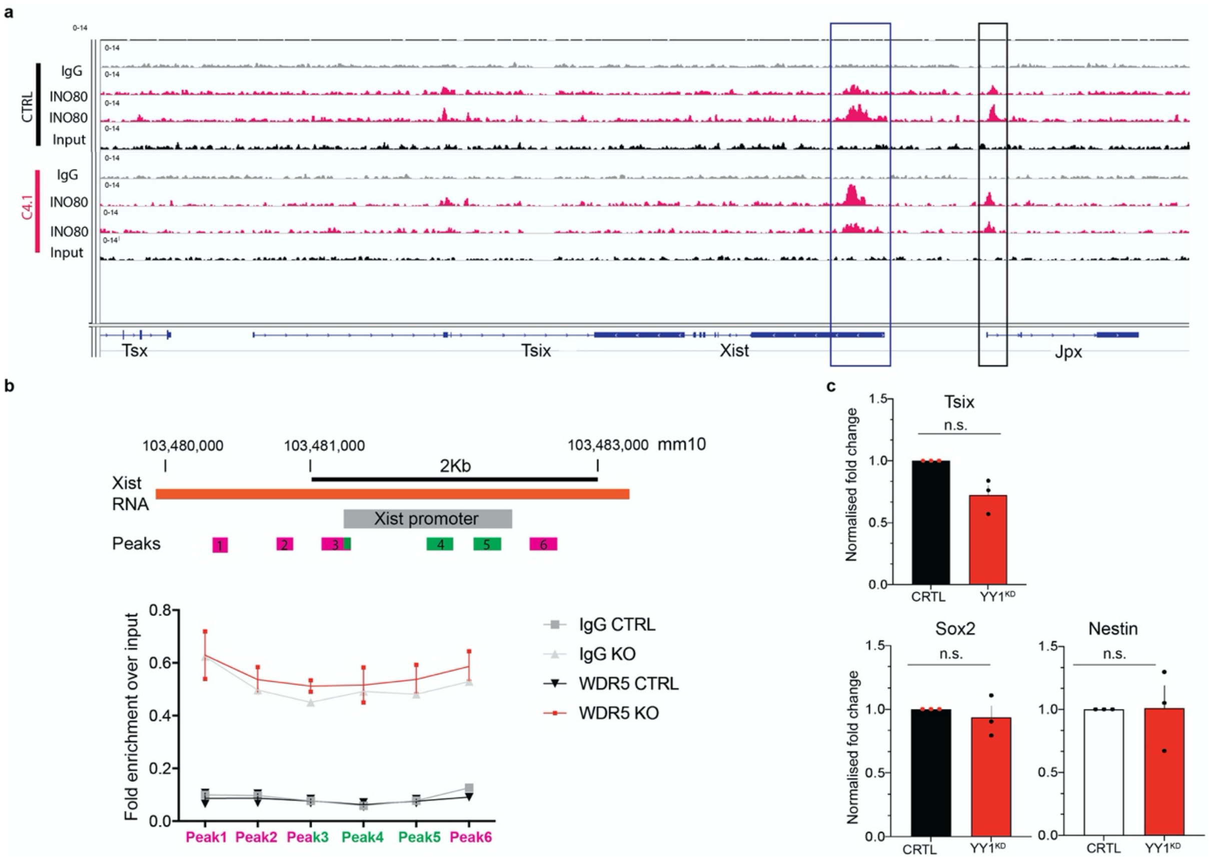

a) INO80 ChIP-seq profiles in parental cell line (Fa2L-S4) and Chd8 KO line (C4.1). Black, undifferentiated conditions; Red, differentiating conditions. Samples are indicated. Xist and Jpx promoters are shown (blue, black frame, respectively) **b)** Top, schematic of Xist promoter and primers used for analysis (peaks 1-6). Bottom, qPCR analysis of Wdr5 Cut & Run qPCR analysis at the Xist promoter. Data from two biological samples are shown. Sample names are shown in the legend. Single points represent independent biological samples. Matched input samples were used as internal normalization control. **c)** CTRL-normalised qRT-PCR analysis of scrambled siRNA (CTRL) and YY1 siRNA (YY1^{KD}) in the Chd8 KO.1 line (KO.1). Genes are indicated. Error bars represent standard deviation of the mean (SEM). Statistical significance was tested by means of two-tailed unpaired t-test (p*≤0.05). Tsix p-value= 0.0746. Data from three experiments are shown. Single points represent independent biological samples. Gapdh was used as internal normalization control.

Fig. S12) *RNA-seq analysis of Chd8 KO cells*

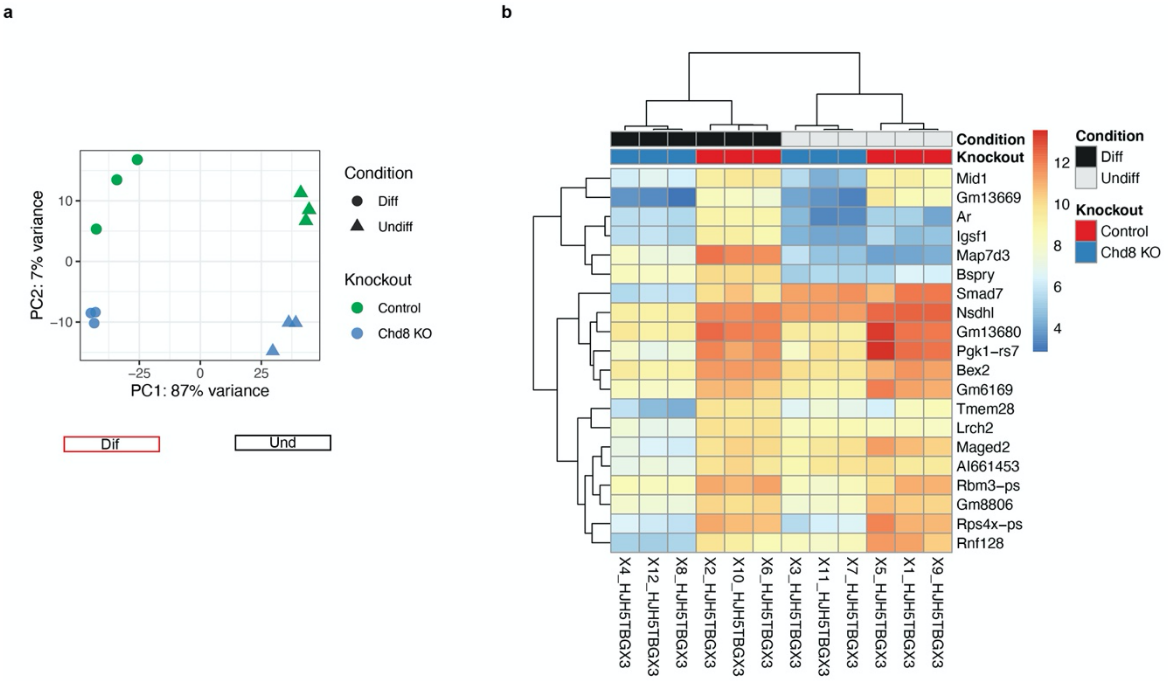

a) PCA plot of differentiated cells vs. undifferentiated cells. In blue the Chd8 KO, in green the control (CTRL). Triangles and circles and indicate undifferentiated and untreated cells (Und - Dif), respectively **b)** Clustered heatmap graph, showing normalized RNA-seq read counts for Chd8 KO vs Controls. The top 20 significantly differentially expressed genes were included in the heatmap.
