# Supplementary material for: Chd8 regulates X chromosome inactivation in mouse through fine-tuning control of Xist expression
Source: Commun Biol. 2021 Apr 15;4:485. doi: 10.1038/s42003-021-01945-1 (PMC8050208; doi:10.1038/s42003-021-01945-1)
Supplement: Supplementary file 3 — Description of Additional Supplementary Files [file 42003_2021_1945_MOESM3_ESM.pdf]

Description of Additional Supplementary Files

File name: Supplementary Data 1

Description: siRNA-mediated Chd8 KD, table of significantly deregulated genes. Gene names and relevant stats are shown.

File name: Supplementary Data 2

Description: shRNA-mediated Chd8 KD, table of significantly deregulated genes (Tab1, Chd8.1; Tab2, Chd8.2). Gene names and relevant stats are shown.

File name: Supplementary Data 3

Description: Chd8 KO, table of significantly deregulated genes. Gene names and relevant stats are shown.

File name: Supplementary Data 4

Description: Chd8 IP mass-spectrometry analysis. Ranked peptides from the used antibodies (see Methods for details).

File name: Supplementary Data 5

Description: Complete list of all primers used in this manuscript.

File name: Supplementary Data 6

Description: Raw data underlying the shown graphs and unprocessed blots.
