# Supplementary material for: Chd8 regulates X chromosome inactivation in mouse through fine-tuning control of Xist expression
Source: Commun Biol. 2021 Apr 15;4:485. doi: 10.1038/s42003-021-01945-1 (PMC8050208; doi:10.1038/s42003-021-01945-1)
Supplement: Supplementary file 10 — Reporting Summary [file 42003_2021_1945_MOESM10_ESM.pdf]

nature research

Corresponding author(s): a.cerase@qmul.ac.uk

Last updated by author(s): Dec. 8th 2020

Reporting Summary

Nature Research wishes to improve the reproducibility of the work that we publish. This form provides structure for consistency and transparency in reporting. For further information on Nature Research policies, see Authors & Referees and the Editorial Policy Checklist .

Statistics

For all statistical analyses, confirm that the following items are present in the figure legend, table legend, main text, or Methods section.

n/a	Confirmed	
☐	☒	The exact sample size (*n*) for each experimental group/condition, given as a discrete number and unit of measurement
☐	☒	A statement on whether measurements were taken from distinct samples or whether the same sample was measured repeatedly
☐	☒	The statistical test(s) used AND whether they are one- or two-sided<br>*Only common tests should be described solely by name; describe more complex techniques in the Methods section.*
☒	☐	A description of all covariates tested
☒	☐	A description of any assumptions or corrections, such as tests of normality and adjustment for multiple comparisons
☒	☐	A full description of the statistical parameters including central tendency (e.g. means) or other basic estimates (e.g. regression coefficient) AND variation (e.g. standard deviation) or associated estimates of uncertainty (e.g. confidence intervals)
☐	☒	For null hypothesis testing, the test statistic (e.g. *F*, *t*, *r*) with confidence intervals, effect sizes, degrees of freedom and *P* value noted<br>*Give P values as exact values whenever suitable.*
☒	☐	For Bayesian analysis, information on the choice of priors and Markov chain Monte Carlo settings
☒	☐	For hierarchical and complex designs, identification of the appropriate level for tests and full reporting of outcomes
☒	☐	Estimates of effect sizes (e.g. Cohen's *d*, Pearson's *r*), indicating how they were calculated

Our web collection on statistics for biologists contains articles on many of the points above.

Software and code

Policy information about availability of computer code

Data collection	NA
Data analysis	NA

For manuscripts utilizing custom algorithms or software that are central to the research but not yet described in published literature, software must be made available to editors/reviewers. We strongly encourage code deposition in a community repository (e.g. GitHub). See the Nature Research guidelines for submitting code & software for further information.

Data

Policy information about availability of data

All manuscripts must include a data availability statement. This statement should provide the following information, where applicable:

- Accession codes, unique identifiers, or web links for publicly available datasets
- A list of figures that have associated raw data
- A description of any restrictions on data availability

Figures 1-5 and S1- 12 have associated raw data or next gen data, included in Supplementary Data 6 file or at the indicated repositories.

Field-specific reporting

Please select the one below that is the best fit for your research. If you are not sure, read the appropriate sections before making your selection.

☒ Life sciences ☐ Behavioural & social sciences ☐ Ecological, evolutionary & environmental sciences

For a reference copy of the document with all sections, see nature.com/documents/nr-reporting-summary-flat.pdf

Life sciences study design

All studies must disclose on these points even when the disclosure is negative.

Sample size	N=2 to 6
Data exclusions	No data was excluded from the analyses
Replication	All experiments were repeated 2 to 3 times
Randomization	NA
Blinding	For slides scoring

Reporting for specific materials, systems and methods

We require information from authors about some types of materials, experimental systems and methods used in many studies. Here, indicate whether each material, system or method listed is relevant to your study. If you are not sure if a list item applies to your research, read the appropriate section before selecting a response.

Materials & experimental systems

n/a	Involved in the study	
☐	☒	Antibodies
☐	☒	Eukaryotic cell lines
☒	☐	Palaeontology
☒	☐	Animals and other organisms
☒	☐	Human research participants
☒	☐	Clinical data

Methods

n/a	Involved in the study	
☐	☒	ChIP-seq
☒	☐	Flow cytometry
☒	☐	MRI-based neuroimaging

Antibodies

Antibodies used	CHD8 Novus biological (NB100-60417) and bethyl (A301-224A), HA ab (H6908) and Vinculin ab (V9264-200UL) ; H3K27me3 Active Motif (#39155, #39536); H3K4me3 antibodies (Active Motif, Cat number 39159); YY1 (H-10): Santa Cruz sc-7341; YY1 Diagenode AB_2793763; WDR5: Diagenode C15310101, INO80 ab was purchased from Abcam (Cat. Number: ab105451)
Validation	The antibodies were validated elsewhere (see company datasheets) or cited publications

Eukaryotic cell lines

Policy information about cell lines

Cell line source(s)	ES cell lines were derived from existing lines. Sources are properly cited in the manuscript.
Authentication	*Describe the authentication procedures for each cell line used OR declare that none of the cell lines used were authenticated.*
Mycoplasma contamination	Cells were routinely tested for mycoplasma contamination
Commonly misidentified lines (See ICLAC register)	No commonly misidentified lines were used

ChIP-seq

Data deposition

☒ Confirm that both raw and final processed data have been deposited in a public database such as GEO.

☒ Confirm that you have deposited or provided access to graph files (e.g. BED files) for the called peaks.

Data access links *May remain private before publication.*	GSE166858 and GSE166859
Files in database submission	*Provide a list of all files available in the database submission.*

Genome browser session
(e.g. UCSC)

Provide a link to an anonymized genome browser session for "Initial submission" and "Revised version" documents only, to enable peer review. Write "no longer applicable" for "Final submission" documents.

Methodology

Replicates	2-3 replicates per ChIP experiment
Sequencing depth	60 to >100M reads per ChIP experiment.
Antibodies	CHD8 Novus biological (NB100-60417) and bethyl (A301-224A), HA ab (H6908) and Vinculin ab (V9264-200UL) ; H3K27me3 Active Motif (#39155, #39536); H3K4me3 antibodies (Active Motif, Cat number 39159); Santa Cruz sc-7341; INO80 ab was purchased from Abcam (Cat. Number: ab105451)
Peak calling parameters	The software MACS2 called peaks with 0.05 as FDR cut-off and consensus peak sets were derived for the duplicated experiments using Bioconductor`s package DiffBind (http://liulab.dfci.harvard.edu/MACS/; https://www.bioconductor.org).
Data quality	Picard tools were used to remove duplicate reads and samTools to filter out reads with mapping quality below two (https://broadinstitute.github.io/picard/). Subsequently, coverage plots were generated with deepTools using a bin size of 50 (or 100b) and RPKM (reads per kilobase per million) normalization.
Software	Analysis has been performed in R (bioconductor) or Python.
