# Supplementary material for: Chd8 regulates X chromosome inactivation in mouse through fine-tuning control of Xist expression
Source: Commun Biol. 2021 Apr 15;4:485. doi: 10.1038/s42003-021-01945-1 (PMC8050208; doi:10.1038/s42003-021-01945-1)
Supplement: Supplementary file 1 — Peer Review File [file 42003_2021_1945_MOESM1_ESM.pdf]

Reviewers' Comments:

Reviewer #1:
Remarks to the Author:
The manuscript by Cerase et al. reports a novel finding that the chromatin remodeler CHD8 regulates X chromosome inactivation by binding at the Xist lncRNA promoter region. The authors use a variety of methods to reduce the amount of CHD8 in a cell line that upon differentiation undergoes XCI. It is unusual that they find a modest decrease in Xist expression upon incomplete knockdown of CHD8, but a strong overexpression of Xist upon severe knockdown or complete knockout of CHD8. Therefore, the authors posit a threshhold effect of CHD8. Furthermore, they find evidence of increased chromatin accessibility within the region of the Xist promoter along with a possible increase in binding of the YY1 transcriptional activator upon knockout of CHD8.

As I stated, the result is novel and interesting. However, I find the results somewhat preliminary, especially the statement included in the title "by competing with transcription factors at the Xist regulatory regions." The manuscript is written carefully to include a multitude of data, but is a bit of a tedious read with the inclusion of so much description of supplementary data. Perhaps, the organization of data into "regular" figures and supplementary figures could be reorganized to clean up the manuscript?

Specific comments are included below:
Major issues that would make manuscript much more convincing
1) The experiments described in Fig. 5D are not convincing. These are critical to the overall conclusion that the mechanism of CHD8 is to compete with transcription factors at Xist. Specifically, the IgG background signal is highly variable in these experiments such that the YY1 signal to noise ratio is very dependent on the IgG "noise." And if the legend to Fig. 5D is correct, all IgG background experiments were done with C4.1 KO cells. To me, the results look promising to support the author's findings, but are not convincing yet.
2) Related to issue #1 above, perhaps the role of YY1 could be established with more certainty if the authors performed a YY1 knockdown experiment in the CHD8 knockout cells?
3) The CHD8 complementation experiment described in Figs 5B,C needs a control. It could simply be transfection with the plasmid vector lacking the chd8 gene? Maybe it was done already and not included? But it should be included.
4) The complicated effect of Xist expression relating to CHD8 knockdown vs knockout is a bit murky. Although I don't consider this essential for the present study, it seems that a much cleaner way to investigate the supposed threshold effect would be to use a tunable (regulatable) knockdown system if one could be set up to effect a wide range of CHD8 reduction.

Minor issues
5) I suggest deletion of Fig. 5A. CHD8 domains are shown already in Fig. 3A, and are not important for the experiments described in Fig. 5.
6) Fig S1 legend needs a description for part C.
7) As mentioned above, the reading of the manuscript is difficult due to the tedious description of much supplementary data. Possibly, this could be shortened (or some of the most "important" supplementary data included as "regular" figures?). For example, I would suggest the reduction of the long description of results from Fig. S4 (pg. 7), and much of that in pg. 8 through half of pg. 9.

Reviewer #2:
Remarks to the Author:
Here Cerase and colleagues investigate a role for the chromatin-remodeller Chd8 in Xist expression and XCI. They provide evidence to suggest that Chd8 may play context-dependent roles in the regulation of Xist expression. Via ChIP-seq, the authors show that Chd8 binds a critical regulatory region within Xist. In undifferntiated ESCs, knockout of Chd8 appears to result in an

increase in Xist expression. In differentiating cells, mild Chd8 knockdown or knockout appears to result in a mild decrease in Xist expression, whereas stronger knockdown of Chd8 or its knockout results in an increase in Xist expression. I do have concerns about some of the data upon which the conclusions were based, but these concerns are addressable. From the authors analyses, it is clear that Chd8 plays a role in the regulation of Xist. With the inclusion of additional data I do believe that the work will constitute an important contribution to the field.

My major concerns revolve around the seemingly contradictory qPCR analyses in the various Xist knockdown experiments.

1) So that readers can better assess the variation in the qPCR data throughout the manuscript, qPCR data points should be plotted individually, overlaid over the bar graphs and error bars. SEM can be misleading. Visually, it under-represents error.

2) Relatedly, in the most stringent qPCR analyses, technical replicates should not be used in significance calculations -- it was unclear whether this was the case here or if the authors used the average of technical replicates in their ANOVA analyses. Because the qPCR data are so important to the conclusions of the study, I do believe that the authors should use the average of technical replicates for significance calculations (ie one data point is considered for each biological replicate).

3) In deriving their conclusions, the authors place a great deal of weight on the qPCR data of Figure 2A, which show less than a one-cycle reduction in Xist expression upon Chd8 siRNA treatment. It is hard for me to be confident in the conclusion of 2A because the observed effect on Xist expression is very small -- again, less than one cycle difference in a qPCR assay -- and because this particular experiment was performed only twice. More biological replicates and a showing of the individual qPCR data points that give rise to the average signal would increase confidence in the findings.

4) Along the same lines, Figure S7C shows no significant difference in Xist levels upon CHD8 knockdown, yet here the authors interpret the trends in the data. While I understand why they interpret the data as they do, more replicates would provide a greater level of confidence in the findings.

The hypothesis that I think the authors should try and rule out by increasing number of replicates is that knockdown of CHD8 has no effect on Xist expression.

5) In Figure 3, the authors should repeat the experiments of C,D, and E in their second CHD KO line, to strengthen the core conclusions.

6) As an alternate means of testing their model, if it were possible to do so, the authors could consider over-expressing CHD8 in WT ESCs, using their CAGGS transgenic construct. Here, the authors would be looking for trends that were opposite from the knockdowns or knockouts. This may be more work than is possible to do at this juncture, but over-expression is nevertheless worth considering.

Minor point:

I am familiar with the data from references 51 and 52 -- however, the interpretation that most of Xist RNA is not polyadenylated may not be correct. The data in ref 52 are also consistent with a polyA tail being tied up in extensive structure at the 3' end of Xist. Indeed, many RNAseq analyses support the existence of full-length Xist that is polyadenylated, and the polyA signal at the very 3' end of Xist matches very strongly with consensus polyA signals.

Reviewer #3:
Remarks to the Author:
The study by Cerase, et al. focuses on determining the role of CHD8, a chromatin remodeler of CHD family, in Xist regulation and initiation of XCI in mouse ESCs. The authors demonstrate that CHD8 binds a number of regulatory elements in Xist and its regulatory circuit, including Tsix and Jpx, which change in their occupancy upon differentiation, when CHD8 levels increase. When CHD8 levels are decreased strongly (80% of d3 levels) or knocked out altogether (KO), Xist RNA increases significantly, and more H3K27me3 signals are detected relative to the control, where about half of cells feature a H3K287me3-marked inactive X. The authors also perform a complementation experiment to demonstrate that these increases are reversed when CHD8 is re-introduced into the homozygous Chd8 knockouts.

Overall, this is an interesting manuscript on an unexplored topic in XCI, develops new key reagents (Chd8 knockout mouse ESCs), and important reference data (CHD8 ChIP-seq, ko ATAC-seq and RNA-seq, CHD8 mass-spec).

Some factors limited enthusiasm for this manuscript in its current form:
1.) The main messages (as summarized above) are somewhat obscured by the unnecessarily detailed discussion of the KD experiments. If the 80% kd , ko, and complementation experiments tell a clear story, why complicate the picture with the "mild" kd experiment? (also why include this in a model figure, and is a model needed/convincing)?

2.) The ATAC-seq and RNA-seq data in the knockout lines are not fully analyzed and important insights are lost as a result. For example:
- how does Xist's regulatory circuit (Jpx, Tsix, Ftx, etc.) respond to Chd8 ko? Can you rule out transcriptional changes in Tsix, Jpx, Ftx as an alternative reason for the increase in Xist?
- is the boost in Xist expression on d3 in Chd8 ko really due to YY1's action? If so, are there other differentially expressed genes in Chd8 ko lines that have CHD8/YY1 co-occupied peaks in wildtype (could use published YY1 mouse ESC chip-seq data to answer this, for example GSE92407)

3.) Is the increase of H3K27me3 in Chd8 ko really due to increasing Xist, or faster (earlier/easier) silencing of the inactive X? Are the more H3K27me3-positive cells, or is the H3K27me3 cloud larger? Not sure I understand this measure or the 2.5-fold increase.

There are some minor comments below, but overall, this is an important study that would benefit from a more in-depth, genome-wide and integrated analysis of the ATAC-seq and RNA-seq data of the CHD8 ko lines, possibly with an eye on including published YY1 peaks to bolster the conclusions about its role. A good revision, and better bioinformatic analyses would greatly improve this already interesting manuscript.

Minor comments:

1.) Supplementary tables listing primer sequences, and analyzed data sets (including from other studies) would be helpful.

2.) How were the qPCR data normalized? Plotting fold-change is confusing as it means two very different things in undifferentiated and d3 cells. Plotting all qPCR data relative to GAPDH or whichever gene was normalized by, would be preferable.

Barts and The London
School of Medicine and Dentistry

Queen Mary University of London
Barts & The London School of Medicine and Dentistry
Blizard Institute - Centre for Genomics and Child Health
4 Newark Street, London E1 2AT, United Kingdom

Dr. Andrea Cerase
Lecturer in Genomics and Epigenetics
Tel: +44(0)20 7882 2594
E-mail: a.cerase@qmul.ac.uk

Dear Sir/Madam,

Please find below a point by point response to the referees' criticisms.

Best wishes,

Andrea Cerase

Reviewers' comments:

Reviewer #1 (Chd8, gene regulation)

The manuscript by Cerase et al. reports a novel finding that the chromatin remodeler CHD8 regulates X chromosome inactivation by binding at the Xist lncRNA promoter region. The authors use a variety of methods to reduce the amount of CHD8 in a cell line that upon differentiation undergoes XCI. It is unusual that they find a modest decrease in Xist expression upon incomplete knockdown of CHD8, but a strong overexpression of Xist upon severe knockdown or complete knockout of CHD8. Therefore, the authors posit a threshhold effect of CHD8. Furthermore, they find evidence of increased chromatin accessibility within the region of the Xist promoter along with a possible increase in binding of the YY1 transcriptional activator upon knockout of CHD8.

As I stated, the result is novel and interesting. However, I find the results somewhat preliminary, especially the statement included in the title "by competing with transcription factors at the Xist regulatory regions." The manuscript is written carefully to include a multitude of data, but is a bit of a tedious read with the inclusion of so much description of supplementary data. Perhaps, the organization of data into "regular" figures and supplementary figures could be reorganized to clean up the manuscript?

Barts and The London
School of Medicine and Dentistry

Queen Mary University of London
Barts & The London School of Medicine and Dentistry
Blizard Institute - Centre for Genomics and Child Health
4 Newark Street, London E1 2AT, United Kingdom

Dr. Andrea Cerase
Lecturer in Genomics and Epigenetics
Tel: +44(0)20 7882 2594
E-mail: a.cerase@qmul.ac.uk

- We thank the referee #1 for his/her overall positive comments. We have re-written and re-organized parts of the manuscript to aid the reader's ease of reading and comprehension – wherever, possible. Our aim of making the paper comprehensible to both specialized and non-specialized readers, however forcibly entails a rather detailed description of the experiments performed.

Specific comments are included below:
Major issues that would make manuscript much more convincing
1) The experiments described in Fig. 5D are not convincing. These are critical to the overall conclusion that the mechanism of CHD8 is to compete with transcription factors at Xist. Specifically, the IgG background signal is highly variable in these experiments such that the YY1 signal to noise ratio is very dependent on the IgG "noise." And if the legend to Fig. 5D is correct, all IgG background experiments were done with C4.1 KO cells. To me, the results look promising to support the author's findings, but are not convincing yet.

-To address these points, we have searched for alternative YY1 antibodies that have shown a good level of enrichment over the background in the literature (i.e. ChIP-qPCR; ChIP-seq experiments). We have found and tested a robust YY1 antibody from Active motive (AB_2793763) and repeated the experiments.
In the new experiment, the antibody specific signal is now significantly higher than the IgG background and shows an increase in YY1 binding to the Xist promoter in Chd8 KO lines **(see amended Fig. 5D).** We believe the results are now clearer and convincing (and in agreement with previous results).

2) Related to issue #1 above, perhaps the role of YY1 could be established with more certainty if the authors performed a YY1 knockdown experiment in the CHD8 knockout cells?

We believe that YY1 is only one of the many players involved in Xist regulation. Knocking down YY1 in Chd8 KO cells, is therefore at best expected to lead to a limited reduction of either Xist level or in the number of cells that initiate XCI, as previously reported[1]. Technical limitations on YY1 depletion linked to its effects on neuronal differentiation[2,3], and the need to ensure continued meaningful comparisons between the samples moreover limit such approaches. In this context we have performed a mild YY1 depletion experiment, and the results have been added in **Fig. 5E**. They confirm that YY1 is important in Xist regulation in both the presence[1] and absence of Chd8. The results moreover suggest that Chd8 and YY1 are 2 players within a complex regulatory system, governing Xist transcription, likely involving multiple redundant players.

3) The CHD8 complementation experiment described in Figs 5B,C needs a control. It could simply be transfection with the plasmid vector lacking the chd8 gene? Maybe it was done already and not included? But it should be included.

Barts and The London
School of Medicine and Dentistry

Queen Mary University of London
Barts & The London School of Medicine and Dentistry
Blizard Institute - Centre for Genomics and Child Health
4 Newark Street, London E1 2AT, United Kingdom

Dr. Andrea Cerase
Lecturer in Genomics and Epigenetics
Tel: +44(0)20 7882 2594
E-mail: a.cerase@qmul.ac.uk

- We have indeed done this experiment but it was not included in the original paper as not pivotal to the reader's interpretation. Upon differentiation, by means of IF we do not observe any significant change of the number of cells initiating XCI in Chd8 KO cells (C4.1) vs Chd8 KO cells complemented with the empty vector. We include the data here for the referees' information.

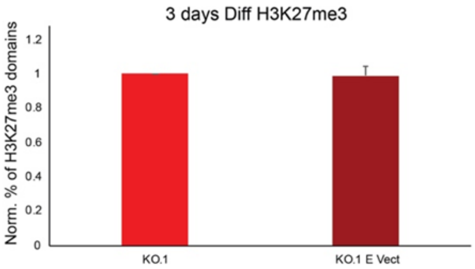

4) The complicated effect of Xist expression relating to CHD8 knockdown vs knockout is a bit murky. Although I don't consider this essential for the present study, it seems that a much cleaner way to investigate the supposed threshold effect would be to use a tunable (regulatable) knockdown system if one could be set up to effect a wide range of CHD8 reduction.

- From previous experience working with tunable vectors, we believe that they often prove to be extremely difficult to set up and we have not therefore attempted this. In order to generate different levels of Chd8 depletion, we have therefore used both different types of KD (si-, sh-RNA mediated) and a generated Chd8 full KO , resulting in a panel of cell lines expressing various levels of CHD8 plus a complete Chd8 depletion. <u>We believe that our approach provides results close in value to those that could be generated by the tunable system suggested by the referee.</u>

Minor issues

5) I suggest deletion of Fig. 5A. CHD8 domains are shown already in Fig. 3A, and are not important for the experiments described in Fig. 5.

- In this figure, we show that Chd8 expression in these experiments shown in this figure is driven by a CAG promoter and not its endogenous promoter (**Fig. 3A**). We believe it is fair to leave this panel for the readers' benefit. We have de-highlighted Chd8 domain to make this clearer.

Barts and The London
School of Medicine and Dentistry

Queen Mary University of London
Barts & The London School of Medicine and Dentistry
Blizard Institute - Centre for Genomics and Child Health
4 Newark Street, London E1 2AT, United Kingdom

Dr. Andrea Cerase
Lecturer in Genomics and Epigenetics
Tel: +44(0)20 7882 2594
E-mail: a.cerase@qmul.ac.uk

6) Fig S1 legend needs a description for part C.

-We have now amended this.

7) As mentioned above, the reading of the manuscript is difficult due to the tedious description of much supplementary data. Possibly, this could be shortened (or some of the most "important" supplementary data included as "regular" figures?). For example, I would suggest the reduction of the long description of results from Fig. S4 (pg. 7), and much of that in pg. 8 through half of pg. 9.

-We have amended and shortened the manuscript whenever possible. However, we believe that a detailed description of the experiments performed supports both the <u>readers' comprehension, especially the non-specialists, and the reproducibility of the experiments performed.</u>

Reviewer #2 lncRNAs, Xist, transcriptional regulation

Here Cerase and colleagues investigate a role for the chromatin-remodeller Chd8 in Xist expression and XCI. They provide evidence to suggest that Chd8 may play context-dependent roles in the regulation of Xist expression. Via ChIP-seq, the authors show that Chd8 binds a critical regulatory region within Xist. In undifferntiated ESCs, knockout of Chd8 appears to result in an increase in Xist expression. In differentiating cells, mild Chd8 knockdown or knockout appears to result in a mild decrease in Xist expression, whereas stronger knockdown of Chd8 or its knockout results in an increase in Xist expression. I do have concerns about some of the data upon which the conclusions were based, but these concerns are addressable. From the authors analyses, it is clear that Chd8 plays a role in the regulation of Xist. With the inclusion of additional data I do believe that the work will constitute an important contribution to the field. My major concerns revolve around the seemingly contradictory qPCR analyses in the various Xist knockdown experiments.

1) So that readers can better assess the variation in the qPCR data throughout the manuscript, qPCR data points should be plotted individually, overlaid over the bar graphs and error bars. SEM can be misleading. Visually, it under-represents error.

- We undertook stringent quality control and statistical analyses to exclude false positive or confounded associations, including confirming that the data did not contain outliers prior to analysis. In light of this, the changes requested by the reviewer are more aesthetic than functional for aiding the reader's interpretation. <u>However, we have replotted all the graphs showing single experimental datapoints as requested.</u> <u>We believe that we can justifiably use SEM as the data has been complemented by rigorous statistical analysis, with no arbitrary exclusion of data.</u>

Barts and The London
School of Medicine and Dentistry

Queen Mary University of London
Barts & The London School of Medicine and Dentistry
Blizard Institute - Centre for Genomics and Child Health
4 Newark Street, London E1 2AT, United Kingdom

Dr. Andrea Cerase
Lecturer in Genomics and Epigenetics
Tel: +44(0)20 7882 2594
E-mail: a.cerase@qmul.ac.uk

Therefore, the reader can always refer to the reported statistics for accurate significance measurements over visual (sometimes misleading) interpretation of average variance (error bars). We also want to stress that the KD conditions were extensively validated before we ran the experiments in the ideal conditions 2 to 3 times (see also below).

2) Relatedly, in the most stringent qPCR analyses, technical replicates should not be used in significance calculations -- it was unclear whether this was the case here or if the authors used the average of technical replicates in their ANOVA analyses. Because the qPCR data are so important to the conclusions of the study, I do believe that the authors should use the average of technical replicates for significance calculations (ie one data point is considered for each biological replicate).

- We only use data points coming from separate biological experiments for statistical testing. Each experiment was performed 2 to 3 times, using at least 2 biological replicates per experiment.
Technical replicates were used only in the qPCR analysis, where each biological sample was run in quadruplicate. The average of these 4 technical replicates was taken to represent the single biological replicate, hence each PCR average corresponds to one biological replicate value. For purposes of visualization 2 to 3 experiments are averaged in the shown graphs (corresponding to 4 to 6 biological (independent) replicates). This information is reported in the figure legend and Material and Methods.
Statistical power and experimental set up were discussed prior the execution of the experiment (AB) and downstream statistical analyses were undertaken in consultation with experienced statisticians who co-authored the paper (AB, SM).

3) In deriving their conclusions, the authors place a great deal of weight on the qPCR data of Figure 2A, which show less than a one-cycle reduction in Xist expression upon Chd8 siRNA treatment. It is hard for me to be confident in the conclusion of 2A because the observed effect on Xist expression is very small -- again, less than one cycle difference in a qPCR assay -- and because this particular experiment was performed only twice. More biological replicates and a showing of the individual qPCR data points that give rise to the average signal would increase confidence in the findings.

- As stated above, the results shown come from multiple biological replicas. We have added more replicates where possible. We are still facing severe COVID-19 lab restrictions.
In this case the results shown come from 3 independent experiments (6 biological samples). Moreover, these results were confirmed, independently in the mild shRNA KD (**Fig. S7**), in the initial work, in independent lines. Please also note that the referee is referring to normalized values, the actual difference in PCR cycles between the samples, is on average 2-3 cycles, not the less than one cycle.

Barts and The London
School of Medicine and Dentistry

Queen Mary University of London
Barts & The London School of Medicine and Dentistry
Blizard Institute - Centre for Genomics and Child Health
4 Newark Street, London E1 2AT, United Kingdom

Dr. Andrea Cerase
Lecturer in Genomics and Epigenetics
Tel: +44(0)20 7882 2594
E-mail: a.cerase@qmul.ac.uk

4) Along the same lines, Figure S7C shows no significant difference in Xist levels upon CHD8 knockdown, yet here the authors interpret the trends in the data. While I understand why they interpret the data as they do, more replicates would provide a greater level of confidence in the f indings. The hypothesis that I think the authors should try and rule out by increasing number of replicates is that knockdown of CHD8 has no effect on Xist expression.

-As above: this experiment comes from 2 experiments, performed with 2 biological replicates per experiments. We do not claim any significant effect of Chd8 KD on Xist transcription in this figure. However, if you consider the single biological points, 3 out of 4 of the Xist qPCR points are significantly lower than the WT control. We believe that it is appropriate in this case describe this as a trend.

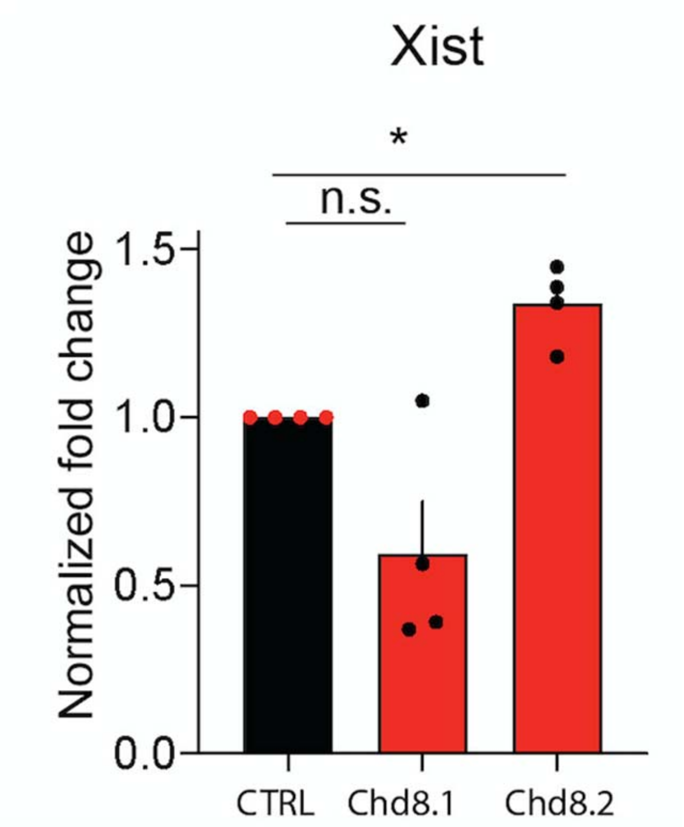

In this graph we show qPCR quantifications in CRTL and Chd8 KD lines. Each point represents a biological experimental datapoint. Bars indicate the standard deviation of the mean (SEM). Statistical significant has been assessed by means of unpaired 2 tailed t-test.

5) In Figure 3, the authors should repeat the experiments of C,D, and E in their second CHD KO line, to strengthen the core conclusions.

Queen Mary University of London
Barts & The London School of Medicine and Dentistry
Blizard Institute - Centre for Genomics and Child Health
4 Newark Street, London E1 2AT, United Kingdom

Dr. Andrea Cerase
Lecturer in Genomics and Epigenetics
Tel: +44(0)20 7882 2594
E-mail: a.cerase@qmul.ac.uk

This experiment was done in the initial phase of this project. We do not believe that it is necessary to add this information in the paper. However, we have added a sentence in the manuscript explaining that similar results were found in the other Chd8 KO line. We also add the original data for the referees' benefit.

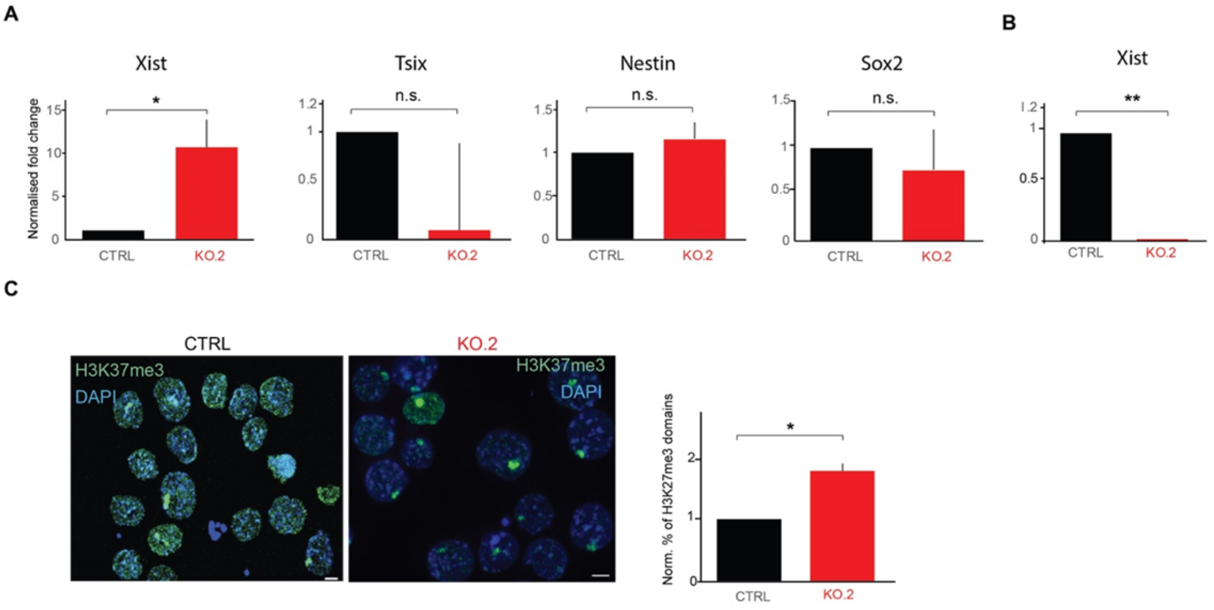

A) qRT-PCR results showing expression levels of Xist, Tsix, Nestin, Sox2 in differentiating Chd8 KO cells (C4.3, KO.2). Xist is strongly up-regulated in Chd8 KO.2 cells whilst cell differentiation is not affected (Nestin, Sox2); CTRL, parental cell line: Fa2L-S4; KO.2. Error bars represent standard deviation of the mean (SEM). Data from three experiments is shown. **B)** Xist qRT-PCR from parental (CTRL) and KO.2 cells are shown, in undifferentiated state. Statistical significance was tested by means of unpaired 2-tailed t-test ($p^* \leq 0.05$; $p^{**} \leq 0.01$). **C)** Representative images of H3K27me3 in CTRL vs. Chd8 KO cells (left) and the scoring of H3K27me3 positive cells (IF) is shown (n>300) (right), bar indicates 10µm. Statistical significance was tested by means of unpaired 2-tailed t-test ($p^* \leq 0.05$; $p^{**} \leq 0.01$). Data from three biological replicas is shown.

6) As an alternate means of testing their model, if it were possible to do so, the authors could consider over-expressing CHD8 in WT ESCs, using their CAGGS transgenic construct. Here, the authors would be looking for trends that were opposite from the knockdowns or knockouts. This may be more work than is possible to do at this juncture, but over-expression is nevertheless worth considering.

Barts and The London
School of Medicine and Dentistry

Queen Mary University of London
Barts & The London School of Medicine and Dentistry
Blizard Institute - Centre for Genomics and Child Health
4 Newark Street, London E1 2AT, United Kingdom

Dr. Andrea Cerase
Lecturer in Genomics and Epigenetics
Tel: +44(0)20 7882 2594
E-mail: a.cerase@qmul.ac.uk

-We are not sure how much we can learn from the suggested experiment and do not consider it as a priority, especially considering the current limitations that we have in the lab, due to the COVID19
pandemic.

Minor point:

I am familiar with the data from references 51 and 52 -- however, the interpretation that most of Xist RNA is not polyadenylated may not be correct. The data in ref 52 are also consistent with a polyA tail being tied up in extensive structure at the 3' end of Xist. Indeed, many RNAseq analyses support the existence of full-length Xist that is polyadenylated, and the polyA signal at the very 3' end of Xist matches very strongly with consensus polyA signals.

-We stated in the manuscript that a large part of Xist is not polyadenylated. We agree however that a significant part of Xist RNA is indeed polyadenylated and have amended the sentence in the text to clarify this.

Reviewer #3 chromatin, ncRNA, XCI

The study by Cerase, et al. focuses on determining the role of CHD8, a chromatin remodeler of CHD family, in Xist regulation and initiation of XCI in mouse ESCs. The authors demonstrate that CHD8 binds a number of regulatory elements in Xist and its regulatory circuit, including Tsix and Jpx, which change in their occupancy upon differentiation, when CHD8 levels increase. When CHD8 levels are decreased strongly (80% of d3 levels) or knocked out altogether (KO), Xist RNA increases significantly, and more H3K27me3 signals are detected relative to the control, where about half of cells feature a H3K287me3-marked inactive X. The authors also perform a complementation experiment to demonstrate that these increases are reversed when CHD8 is re-introduced into the homozygous Chd8 knockouts.
Overall, this is an interesting manuscript on an unexplored topic in XCI, develops new key reagents (Chd8 knockout mouse ESCs), and important reference data (CHD8 ChIP-seq, ko ATAC-seq and RNA-seq, CHD8 mass-spec).

Some factors limited enthusiasm for this manuscript in its current form:
1.) The main messages (as summarized above) are somewhat obscured by the unnecessarily detailed discussion of the KD experiments. If the 80% kd , ko, and complementation experiments

Barts and The London
School of Medicine and Dentistry

Queen Mary University of London
Barts & The London School of Medicine and Dentistry
Blizard Institute - Centre for Genomics and Child Health
4 Newark Street, London E1 2AT, United Kingdom

Dr. Andrea Cerase
Lecturer in Genomics and Epigenetics
Tel: +44(0)20 7882 2594
E-mail: a.cerase@qmul.ac.uk

tell a clear story, why complicate the picture with the "mild" kd experiment? (also why include this in a model figure, and is a model needed/convincing)?

-We believe that it is fair and correct showing all the results that we had to the readers, who can then autonomously interpret the data.

2.) The ATAC-seq and RNA-seq data in the knockout lines are not fully analyzed and important insights are lost as a result. For example: how does Xist's regulatory circuit (Jpx, Tsix, Ftx, etc.) respond to Chd8 ko? Can you rule out transcriptional changes in Tsix, Jpx, Ftx as an alternative reason for the increase in Xist?

-We have performed the analysis as suggested by the referee for all XIC genes. Despite the changes at chromatin level for some genes (i.e. ATAC-seq analysis), we do find a significant downregulation of Chic1 and Rnf12 and Ftx, both Xist activators, suggesting that Xist upregulation is unlikely linked to the activity of Xist regulators.

is the boost in Xist expression on d3 in Chd8 ko really due to YY1's action? If so, are there other differentially expressed genes in Chd8 ko lines that have CHD8/YY1 co-occupied peaks in wildtype (could use published YY1 mouse ESC chip-seq data to answer this, for example GSE92407)

- We have downloaded the data from the work suggested by the referee (ES cells grown in 2i conditions, GSE92407[4] and, not surprisingly we discovered a large overlap between the YY1 and Chd8 peaks, with 75% of Chd8 peaks also bound by YY1. Given the very high overlap, particularly in promoter regions (95%) between these two datasets, we also report a large number of YY1 peaks in promoters of genes found to be differentially expressed.

Here is a summary of the requested analysis, we now report this analysis in the discussion. We have added relevant information in the manuscript.

Summary of the analysis of the cells in 2i conditions:

- Out of a total of 24,464 Chd8 peaks 18,117 overlap YY1 peaks (~75%)
- Chd8 peaks map in the promoter region of 6,713 of the genes included in the differential expression analysis (defined by up to 2kb upstream of the TSS).
- 6,378 of these also have a YY1 peak (95%).
- Of the 2,484 differentially expressed genes in the Chd8 knockout (differentiated) 708 promoters are bound by Chd8 and of those 638 (90%) also by YY1.

Note: while the recommended work (GSE92407[4]) also contained data from differentiating cells, their differentiating conditions were profoundly different and did not allow any meaningful comparison.

Barts and The London
School of Medicine and Dentistry

Queen Mary University of London
Barts & The London School of Medicine and Dentistry
Blizard Institute - Centre for Genomics and Child Health
4 Newark Street, London E1 2AT, United Kingdom

Dr. Andrea Cerase
Lecturer in Genomics and Epigenetics
Tel: +44(0)20 7882 2594
E-mail: a.cerase@qmul.ac.uk

3.) Is the increase of H3K27me3 in Chd8 ko really due to increasing Xist, or faster (earlier/easier) silencing of the inactive X? Are the more H3K27me3-positive cells, or is the H3K27me3 cloud larger? Not sure I understand this measure or the 2.5-fold increase.

-A 2.5-fold increase over the parental baseline indicates that more cells have started XCI at day 3 of differentiation in the KO cell line. We do not have any indication of enhance differentiation by qRT-PCR or RNA-seq. We have clarified this now in the text.

There are some minor comments below, but overall, this is an important study that would benefit from a more in-depth, genome-wide and integrated analysis of the ATAC-seq and RNA-seq data of the CHD8 ko lines, possibly with an eye on including published YY1 peaks to bolster the conclusions about its role. A good revision, and better bioinformatic analyses would greatly improve this already interesting manuscript.

-This analysis has been done and incorporated in the last version of this manuscript.

Minor comments:

1.) Supplementary tables listing primer sequences, and analyzed data sets (including from other studies) would be helpful.

We have now include this information in **Supplementary Table 5** (all primers used). Datasets analyzed and antibody used have been included in the Material and Methods.

2.) How were the qPCR data normalized? Plotting fold-change is confusing as it means two very different things in undifferentiated and d3 cells. Plotting all qPCR data relative to GAPDH or whichever gene was normalized by, would be preferable.

-We plot changes over undifferentiated conditions for selected differentiation markers as this helps the reader to understand that the cells where properly differentiated (or gene silencing efficiency was not affected). Most of the other graphs have been normalized to the parental control line or CTLR (scrambled siRNA), to aid the reader's interpretation of the data.

References

Barts and The London
School of Medicine and Dentistry

Queen Mary University of London
Barts & The London School of Medicine and Dentistry
Blizard Institute - Centre for Genomics and Child Health
4 Newark Street, London E1 2AT, United Kingdom

Dr. Andrea Cerase
Lecturer in Genomics and Epigenetics
Tel: +44(0)20 7882 2594
E-mail: a.cerase@qmul.ac.uk

1 Makhlouf, M. *et al.* A prominent and conserved role for YY1 in Xist transcriptional activation. *Nature communications* **5**, 4878, doi:10.1038/ncomms5878 (2014).

2 Beagan, J. A. *et al.* YY1 and CTCF orchestrate a 3D chromatin looping switch during early neural lineage commitment. *Genome research* **27**, 1139-1152, doi:10.1101/gr.215160.116 (2017).

3 Zurkirchen, L. *et al.* Yin Yang 1 sustains biosynthetic demands during brain development in a stage-specific manner. *Nature communications* **10**, 2192, doi:10.1038/s41467-019-09823-5 (2019).

4 Atlasi, Y. *et al.* Epigenetic modulation of a hardwired 3D chromatin landscape in two naive states of pluripotency. *Nat Cell Biol* **21**, 568-578, doi:10.1038/s41556-019-0310-9 (2019).

Reviewers' Comments:

Reviewer #1:
Remarks to the Author:
In this revised manuscript, the authors have addressed satisfactorily the scientific issues raised by this reviewer from the initial version. New experiments were completed for Figure 5, Figure 3A was modified slightly, and the legend to Figure S1 was modified. Also, the authors showed that the control experiment suggested for Figure 5 was completed previously.

This reviewer notes that the manuscript remains a tedious read, but recognizes that this comment is one of style that is best left to the journal editors. In no way does this comment affect the quality and conclusions of the science.

Reviewer #2:
Remarks to the Author:
I thank the authors for responding to my concerns and I would support publication so long as the editors conditions have been met.

Reviewer #3:
Remarks to the Author:
The authors have clarified or addressed most of my concerns regarding the presentation of their results. However, the computational analysis of published YY1 data and comparison the data presented herein, was to ascertain whether the interplay of CHD8 and YY1 observed at Xist could be generalized across the rest of the genome, or whether there were other features to predict whether genes would increase or decrease in the absence of CHD8. At minimum that would require showing whether YY1/CHD8 co-occupied promoters increase or decrease in expression, as posited here. Nonetheless, I appreciate that was not the main point of this paper.

A second point of contention are the mild K.D. experiments which stand in contrast to the K.O. results, and could have resulted from shRNA-related off-target or subtle differentiation-related effects. In my view, these results muddy the otherwise clear conclusions from the CHD8 K.O. lines, and I see little value in including them. Nonetheless, it should be the authors decision whether they want to include these K.D. data.

Finally, it was surprising the very significant loss of Tsix expression wasn't discussed in more detail, though I suppose that's because a Tsix-termination line was used, which may rule out an CHD8 effect on Xist via Tsix? Please state so in the final manuscript if so.

A final revision of the paper should probably include a more in-depth presentation of the CHD8 differential expression results. As my other core concerns have been addressed in the revised manuscript, it's not necessary for me to review it again, and I support publication.

Is the GEO deposition in progress? There should be an accession number (not GEOXXYY) even if it's still in embargo until publication.

Referee #1 (nuclear membrane proteins)

In the opinion of this reviewer, the authors have addressed the concerns and the data are consistent with the original conclusions.

- We thank **Referee #1** for recommending our paper for publication. We believe that the data contained in this manuscript is important for the laminopathy community and for the whole scientific community.

One minor edit: I did not find a statement in the revised main text regarding the possible problem with LBR localization (#1 of rebuttal letter). This statement should be included somewhere in the first paragraph of the main text.

- This statement was already present in the submitted version. In the discussion section of the manuscript, we wrote: "As a potential caveat, we cannot exclude that Lbr localization to the inner nuclear membrane might be affected in our mutants. However, recent reports have shown that a full N-terminal mutation is capable of nuclear membrane localization via its transmembrane domains".

I do not need to see the ms again.

Reviewer #2 (XCI specialist):

The authors of the manuscript titled "A N-terminal truncation of the lamin B receptor (Lbr) gene recapitulates the phenotypes of the Pelger-Huet anomaly and shows minor X chromosome inactivation defects" have taken the majority of comments from the first round of review into consideration and have addressed the majority of issues raised by the reviewers. Especially the effect of the presented N-terminal truncation of LBR on random XCI in vivo and in vitro has been addressed. Thus, I would recommend to consider the re-submitted manuscript for publication. Please find below my comments to this new version of the manuscript.

General comments:

As recommended in the first round of review the authors have extended their analysis of XCI phenotypes. The reported results contradict the observation from Chen et al., 2016 and are more in line with the results reported in Nesterova et al., 2019. The authors show in vivo and in ESCs derived from NT-KO mutants (in vitro), that the LBR NT is not required for initiation of XCI. I recommend discussing the results accordingly with a clean separation of expected XCI initiation and maintenance phenotypes.
The LBR NT supposedly binds Xist. The interaction of Xist with LBR has been proposed to be "required for Xist-mediated silencing" (Chen et al., 2016). Apparently, this interaction is not required in vivo and in vitro in the systems the authors looked at.
The only X chromosome related phenotype observed by the authors (in the systems they looked at) is a shift towards higher distances from the nuclear lamina. This change in nuclear

organization is interesting, but was not linked to gene expression changes of normally silenced genes on the X in vivo and in vitro. I am not sure this change would qualify as a 'minor X chromosome inactivation defect' as XCI (i.e. gene silencing) does not appear to be affected in vitro and in vivo.

-While we agree with Referee #2, in the regards to general comments, we do not fully agree in relation with the affirmation that the data included in our paper is closer to Nesterova et al. 2019 than Chen et al, 2020. Nor that our work contradicts the work presented in Chen et al.
The work by Nesterova et al, is mostly done using inducible Xist systems, while we relied on endogenous and physiological Xist expression during differentiation. Furthermore, while we have removed the Xist interacting regions at the protein level (LBR NT-KO), Nesterova et al, have abolished (or diminished) Xist binding by means of Xist deletions and full protein KD/KO. Finally, Nesterova et al., uses a different system to assess gene silencing on a chromosome-wide level, while we only tested selected genes, based on published literature. <u>We believe that these two systems are not directly comparable.</u>
In regards to the data presented by Chen et al, we report an increased Xi-lamina distance in our NT-KO mutant, and minor silencing effects. This is in agreement with the work of Chen et al.
In brief, we believe that our work, while it is in agreement with Nesterova et al. 2019, does not disprove the work published in Chen et al, 2016.

Line 111-113:
The role of LBR in the initiation of random XCI has been the subject of debate. I would recommend mentioning this here. (Nesterova, T. B. et al. Systematic allelic analysis defines the interplay of key pathways in X chromosome inactivation. Nat Commun 10, 3129 (2019).

-We have included a sentence and this citation in the text, in relation to Lbr role in Xist-mediated gene silencing.

Line 127-133/Figure 3B:
The authors claim in the text that there was no major difference in the expression of the tested silenced and escaping genes by qRT-PCR analysis. While based on Figure 3 I would agree for the majority of normally silenced genes, it appears as if there is more silencing of escaping genes such as Kdm5c (Jarid1c) or MeCP2 (both facultative escapees) in the NT-KO. Both are marked as non-significant, though they are expressed at only 50% of WT levels with rather short error bars. This is counterintuitive as one would expect higher expression in the NT-KO situation if there was a silencing defect. There could be something interesting here with regard to escaping genes.

-We agree with referee N2 and we are planning to follow-up this by means of scRNA-seq and scDAM-ID analysis. As reported in the paper, we believe that most of gene-expression changes are linked to the abnormal repositioning of the nuclear heterochromatin in our mutants cells/animals. The figure has been revised and amended.

Line 127-133/Figure 3/Supp. Figure 4:
One thing that is commonly assessed in this type of experiment are differentiation dynamics. Comparing the fraction of cells with an inactive X or gene silencing dynamics only makes sense if it can be assured that two cell lines differentiate with similar dynamics. This can easily be done by testing expression/downregulation of pluripotency markers by e.g. qPCR.

-We routinely follow cell differentiation in all our experiments. The requested analysis was done but not included in the manuscript. We have now added this information in **Fig. S4C.**

Line 140-143:
Based on what the authors present in the results part LBR is not essential for XCI initiation in vivo. Based on the qPCR for silenced genes (Figure 3B) and the fraction of cells that initiated random XCI (Sup. Fig. 4), LBR is also not essential for initiation of random XCI in ESCs derived from NT-KO homozygous embryos. The greater distance to the lamina of the inactive X in NT-KO cells appears to be rather a secondary effect in heterochromatin organization than "the LBR playing a role in random XCI". I would recommend rephrasing that. The phenotype might be due to heterochromatin organizing more in the nuclear interior similar to what has been observed for silenced olfactory receptor genes in LBR+ vs LBR- cells (Clowney, E. J. et al. Nuclear aggregation of olfactory receptor genes governs their monogenic expression. Cell 151, 724–737 (2012)).

- We agree with ref. N2 and rephrased that sentence accordingly and cited the suggested paper.

Line 149-157:
I strongly disagree with this discussion point. I would limit the discussion here to the role of LBR in gene regulation and development. As there is no clear XCI phenotype, I don't see the necessity to invoke XCI here, to explain homozygous "survivors". I am not sure there is a need to disentangle development and XCI, as the authors do not report a female-specific effect on viability or X-linked gene mis-regulation in females/differentiating female ESCs.
Their in vivo work and the results on the ESC derived from their N-terminal LBR mutant mouse embryos clearly show that LBR NT is not required for initiation of XCI. Also, there is no clear mis-regulation of silenced genes in LBR mutant animals (RNAseq in vivo and qPCR in differentiating ESCs).

-We believe that it is possible that our analysis did not capture minor XCI defects in our LBR mutation in early development. Indeed, we reported increased embryonic lethality when the mutation was transmitted by the father - <u>potentially suggesting defects in the imprinted X inactivation process</u>. However, we do not report differences between the sexes. We would like to keep this possibility open for discussion and prompt future investigations. We are planning to follow-up on this analysis, using more sensitive approaches.

Minor comments:

Line 87/88: The authors mention the essential role of Xist for random XCI and Rnf12/Rlim in imprinted XCI in vivo. Proper Xist function on the paternal X is of course also essential for imprinted XCI.

-We have now clarified this as well.

Line 100: The authors refer to "these regulatory defects" without specifying beforehand what those are.

-This has been clarified in the text.

Line 158: N-terminus (a spelling mistake)

-This has been amended.

To Figures 3A:
Maybe a 'zoom-in' to representative examples would help to illustrate the distance shift.

-This has been done, we thank the referee for the very useful and improving suggestion.

To Figure 3A/S4B:
Please specify the number of analyzed cells per experiment/number of experiments in the figures or figure legends, respectively.

-The figure legend has been amended accordingly.

Reviewer #3 (Mass spectrometry expertise):

The authors have generated a novel mouse model that presents for Pelger-Huet Anomaly without additional defects associated with Greenberg Dysplasia. While I anticipate this is an important model and fills an important need, the characterization of the gene product (that is described as the key player) is still lacking.
I appreciate the author's effort to provide a description of the methods used in their mass spectrometry characterization. As I anticipated, the authors used trypsin for digestion of the truncated Lbr and subsequent proteomics analysis. I still maintain that the sequence coverage is too low (even for membrane proteins) and does not support the conclusions they have made. This result can be improved by:

1. Obtaining an intact mass measurement by MALDI or ESI. This would provide the most definitive evidence for the truncated Lbr gene product and rule out any anomalies due to heterogeneity in the anticipated gene product (e.g. proteins from alternative translation start sites).

-As mentioned before, the antibody used in these experiments has been discontinued and we cannot repeat these experiments (i.e. Lbr-IP). As our mutant protein is very diluted, we will not be able to pick up (or enrich for) Lbr mutant peptides without any IP.
As discussed in the previous rebuttal, the used antibodies, cannot detect the reported 3' alternative splicing products. We are not aware of 5' Lbr alternative splicing products. We cannot predict from publicly-available ESTs databases any 5' alternative splicing product compatible both with the WB and Mass-Spectrometry data. Crucially, if the observed band in our mutant was the product of 5' alternative splicing (bypassing our mutation), its protein level should be expressed at a comparable level to the matched WT control, and not severely diminished as we report.

2. Using a more promiscuous protease such as chymotrypsin in the IP-MS experiments to improve sequence coverage.

-As above.

The authors have indicated that they are no longer capable of carrying out additional experiments because they ran out of suitable antibodies for immunoprecipitation, which brings me to a third recommendation:
3. Manually search the MS data for peptides that were missed by the analysis software. Based on the expected sequence of truncated Lbr, the authors can expect to generate ~33 unique tryptic peptides, however 13 of those peptides have residues below the threshold for analysis (<7 amino acids). The authors may consider going through the raw data to determine if the low sequence coverage can be attributed to "missed" data. However, my suspicion is that digestion efficiency was low considering there was opportunity to detect up to 20 unique peptides under these conditions and the plot in Figure S1 appears to indicate ~5.

Our analysis revealed a total of 54 peptides in total across the replicas, with different degree of confidence (indicated in the figure legend), 29 of which high-confidence unique peptides. While the number of unique peptides recovered in our NT-KO samples do not provide full coverage, this is most likely due to the very low abundance of the protein in the NT-KO condition rather than an incomplete digestion. This is also fully supported by the WB data. We want to stress that the mass-spectrometry analysis has been performed at one of the best facilities in Europe (the EMBL Proteomic Core Facility). This facility is internationally renowned and it has an outstanding-quality track record.

The WB analysis that the authors claim to be complementary to the MS results also comes with discrepancies that don't strengthen the authors' conclusions. For example, the additional bands in the WT (using the SantaCruz antibody) have been attributed to an uncharacterized degradation product. In the NT-KO, the very faint band attributed to the truncated Lbr very closely resembles the molecular weight of the degradation product. The resolution offered by gel electrophoresis is not sufficient to classify these bands as different proteins and so without further characterization of the "degradation product" the claim that the faint band is the target protein seems to be a convenient conclusion rather than a supported one.

- We do not state that the truncated protein or the degradation product are necessarily of different size. They are indeed in a very similar range as clearly stated in the manuscript. We used gradient SDS-PAGE gels to detect any difference in MW and gel mobility. However, we had to load an excess of protein in NT-KO lanes in order to detect the mutated, truncated proteins, which makes it very difficult to assess with absolute certainty the exact molecular weight of the truncated protein. For these reasons, **all the analysis of the protein products, has been accompanied by rigorous and parsimonious analysis of the matched DNA and RNA sequences**.
Finally, we believe that the degradation product is likely to represent a significant deletion of the cytosolic LBR N-terminal part. As our mutation also deletes a significant part of LBR- N-terminus, **it is expected that the two bands might run at a very similar MW**. The size difference between the degradation product and our mutant (if any) is completely irrelevant for the significance of this work. As stated above, an alternative splicing variant is extremely unlikely and not in line with the underlying genetic mutation and the produced RNA (mRNA-seq data). We do not find experimental support for a 5' alternative-splicing product.

The authors have convincingly predicted the likely translation start sites, however the characterization of the resulting gene product is ultimately the most important result. The current WB and low sequence coverage are not adequate for the author's claims about the molecular composition of their truncation. The results have important implications for the generation of this novel mouse model however the characterization is lacking and severely undercuts the importance of the claims.

- We do agree with the referee that a fuller characterisation would add more importance to the mouse model and we do plan to follow this up.
